# Studies on the substrate specificity of a GDP-mannose pyrophosphorylase from *Salmonella enterica*

**DOI:** 10.3762/bjoc.8.136

**Published:** 2012-08-01

**Authors:** Lu Zou, Ruixiang Blake Zheng, Todd L Lowary

**Affiliations:** 1 Alberta Glycomics Centre and Department of Chemistry, The University of Alberta, Edmonton, AB T6G 2G2, Canada

**Keywords:** chemoenzymatic synthesis, kinetics, methylation, pyrophosphorylase, sugar nucleotide

## Abstract

A series of methoxy and deoxy derivatives of mannopyranose-1-phosphate (Man*p*-1P) were chemically synthesized, and their ability to be converted into the corresponding guanosine diphosphate mannopyranose (GDP-Man*p*) analogues by a pyrophosphorylase (GDP-ManPP) from *Salmonella enterica* was studied. Evaluation of methoxy analogues demonstrated that GDP-ManPP is intolerant of bulky substituents at the C-2, C-3, and C-4 positions, in turn suggesting that these positions are buried inside the enzyme active site. Additionally, both the 6-methoxy and 6-deoxy Man*p*-1P derivatives are good or moderate substrates for GDP-ManPP, thus indicating that the C-6 hydroxy group of the Man*p*-1P substrate is not required for binding to the enzyme. When taken into consideration with other previously published work, it appears that this enzyme has potential utility for the chemoenzymatic synthesis of GDP-Man*p* analogues, which are useful probes for studying enzymes that employ this sugar nucleotide as a substrate.

## Introduction

**Figure 1:**
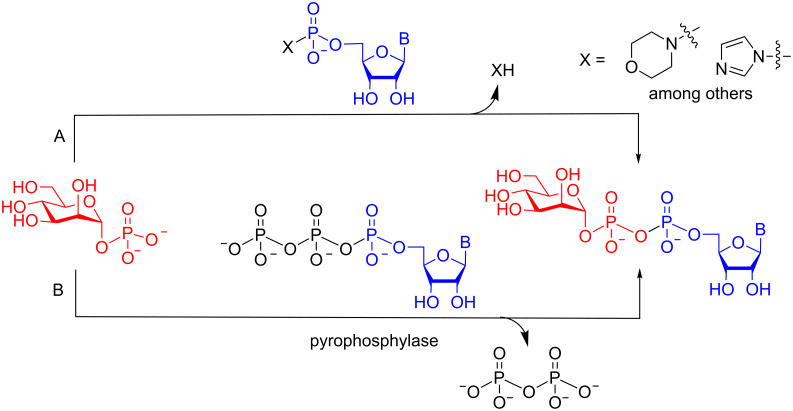
(A) Conventional approach for the chemical synthesis of sugar nucleotides from sugar 1-phosphates; (B) enzymatic conversion of sugar 1-phosphates into sugar nucleotides.

Modified sugar nucleotide analogues are valuable probes to study glycosyltransferases and other enzymes that use these activated glycosylating agents as substrates [1–5]. The synthesis of natural and non-natural sugar nucleotides is therefore a topic of continuing interest [6]. The classical method for chemically synthesizing sugar nucleotides involves the preparation of a sugar 1-phosphate derivative followed by its coupling to an activated nucleoside monophosphate to form the key pyrophosphate moiety (Figure 1A) [7]. In general, the yield of this process is low, and the purification of the product can be tedious; hence, the development of new methods to prepare sugar nucleotides remains an area of active research [6]. Although improved chemical methods have been developed [8–13], another attractive strategy is to employ a chemoenzymatic approach, in which a synthetic sugar 1-phosphate derivative is converted to the sugar nucleotide by a pyrophosphorylase (Figure 1B) [14–15]. This approach is increasingly used for the synthesis of sugar nucleotides, but a limitation is that the specificity of the pyrophosphorylase must be sufficiently broad to recognize the synthetic sugar 1-phosphate derivative. However, some of these enzymes have been demonstrated to have broad specificity, or can be engineered to have broad specificity, with regard to both the sugar 1-phosphate and nucleotide substrates [16–19].

**Figure 2:**
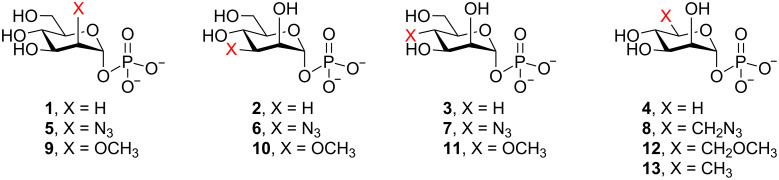
Structures of the Man*p*-1P derivatives (**1**–**8**) previously shown [24–25] to be substrates for *S. enterica* GDP-ManPP and analogues **9**–**13** studied in this paper.

As part of a larger study on the specificity of mannosyltransferases involved in mycobacterial glycan biosynthesis [20–22], we had the need for a panel of singly deoxygenated and methylated guanosine diphosphosphate mannopyranose (GDP-Man) derivatives. In developing a strategy for the synthesis of these compounds, we chose to take advantage of a GDP-mannose pyrophosphorylase (GDP-ManPP) from *Salmonella enterica* [23], which had previously been shown to have a relaxed specificity for the sugar 1-phosphate moiety [24–25]. In particular, it has been shown that the enzyme will accept mannopyranosyl 1-phosphate (Man*p*-1P) derivatives deoxygenated at C-2, C-3 and C-4 (**1**–**3**, Figure 2), as well as a substrate lacking the hydroxymethyl group at C-5 (**4**) [24]. A series monoazido derivatives (**5**–**8**) were also shown to be substrates [25]. To further probe the potential of this enzyme for the chemoenzymatic synthesis of modified GDP-Man*p* derivatives, we describe here the preparation of all four singly methylated Man*p*-1P analogues **9**–**12**, as well as the 6-deoxy-Man*p*-1P derivative **13**, and an initial evaluation of their ability to serve as a substrate for *S. enterica* GDP-ManPP.

## Results and Discussion

### Synthesis of 2-methoxy derivative **9**

**Scheme 1:**
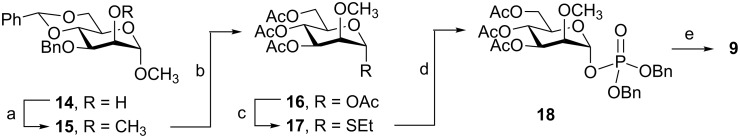
Reagents and conditions: (a) CH_3_I, NaH, DMF, 80%; (b) Ac_2_O–HOAc–H_2_SO_4_, 35:15:1, 81%; (c) EtSH, BF_3_·OEt_2_, CH_2_Cl_2_, 65%; (d) HO-P(O)(OBn)_2_, NIS, AgOTf, CH_2_Cl_2_, 84%; (e) (i) H_2_, Pd(OH)_2_–C, toluene, Et_3_N, pyridine; (ii) CH_3_OH–H_2_O–Et_3_N, 5:2:1, 92%.

The synthesis of sugar 1-phosphate **9** containing a methyl group at O-2 commenced from 3-*O*-benzyl-4,6-*O*-benzylidene-α-D-mannopyranoside **14** [26] as illustrated in Scheme 1. Methylation of the alcohol under standard conditions proceeded in 80% yield affording **15**. The benzylidene protecting group was cleaved, together with the methyl glycoside, by acetolysis giving the tetra-*O*-acetylated compound **16** in 81% yield. This glycosyl acetate was converted to the corresponding thioglycoside (**17**), which was, in turn, coupled with dibenzyl phosphate under NIS–AgOTf activation conditions, providing compound **18** in 55% yield over two steps from **16**. The anomeric stereochemistry in **18** was confirmed by the magnitude of the ^1^*J*_C1,H1_, which was 177.9 Hz, consistent with α-stereochemistry as described earlier by Timmons and Jakeman for rhamnopyranosyl phosphates [27]. In the other phosphorylation reactions reported in this paper, the anomeric stereochemistry was determined in an analogous manner. Compound **18** was then deprotected in two steps, namely catalytic hydrogenolysis and then, without further purification, treatment with a mixture of CH_3_OH–H_2_O–Et_3_N 5:2:1 to remove the acetyl groups. This series of reactions gave 2-methoxy Man*p*-1P analogue **9** in 92% overall yield from **18**.

### Synthesis of 3-methoxy derivative **10**

**Scheme 2:**
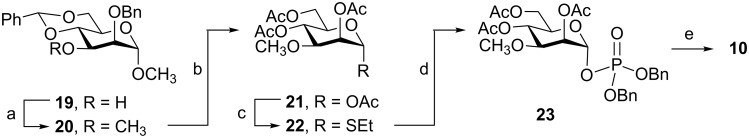
Reagents and conditions: (a) CH_3_I, NaH, DMF, 76%; (b) Ac_2_O–HOAc–H_2_SO_4_, 35:15:1, 65%; (c) EtSH, BF_3_·OEt_2_, CH_2_Cl_2_, 52%; (d) HO-P(O)(OBn)_2_, NIS, AgOTf, CH_2_Cl_2_, 75%; (e) (i) H_2_, Pd(OH)_2_–C, toluene, Et_3_N, pyridine; (ii) CH_3_OH–H_2_O–Et_3_N, 5:2:1, 67%.

The preparation of the 3-methoxy Man*p*-1P analogue **10** followed a route similar to that used for the synthesis of **9** (Scheme 2). Methyl 2-*O*-benzyl-4,6-*O*-benzylidene-α-D-mannopyranoside (**19**) [26] was first methylated giving **20** and then converted into glycosyl acetate **21** in 49% yield over the two steps. Subsequent thioglycosylation provided a 52% yield of **22**. The protected dibenzyl phosphate **23** was next formed by the NIS–AgOTf promoted glycosylation of dibenzyl phosphate with **22**, which afforded the desired compound, **23**, in 75% yield. Hydrogenolysis of the benzyl groups and deacylation led to the formation, in 67% yield, of Man*p*-1P derivative **10**.

### Synthesis of 4-methoxy derivative **11**

**Scheme 3:**
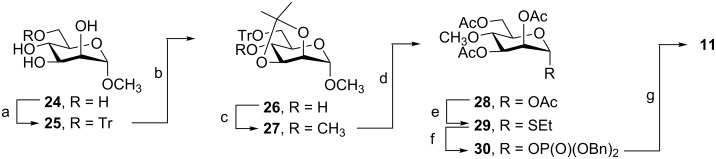
Reagents and conditions: (a) TrCl, DMAP, pyridine, 85%; (b) DMP, *p*-TsOH, 76%; (c) CH_3_I, NaH, DMF, 91%; (d) Ac_2_O–HOAc–H_2_SO_4_, 35:15:1, 55%; (e) EtSH, BF_3_·OEt_2_, CH_2_Cl_2_, 70%; (f) HO-P(O)(OBn)_2_, NIS, AgOTf, CH_2_Cl_2_, 80%; (g) (i) H_2_, Pd(OH)_2_–C, toluene, Et_3_N, pyridine; (ii) CH_3_OH–H_2_O–Et_3_N, 5:2:1, 70%.

As illustrated in Scheme 3, the synthesis of the 4-methoxy Man*p*-1P analogue **11** started by treatment of methyl α-D-mannopyranoside (**24**) with trityl chloride in pyridine. The product, **25**, was then converted to the isopropylidene acetal **26** in 65% overall yield from **24**. The hydroxy group in **26** was methylated under standard conditions (CH_3_I, NaH) to give the 4-methoxy analogue **27** in 91% yield. Acetolysis of **27** to the corresponding glycosyl acetate **28**, followed by reaction with ethanethiol and BF_3_**·**OEt_2_, yielded thioglycoside **29**, in a modest 39% yield from **27** over two steps. This compound was then converted to **11**, in 56% yield, as outlined above, by successive phosphorylation and deprotection.

### Synthesis of 6-methoxy derivative **12**

**Scheme 4:**
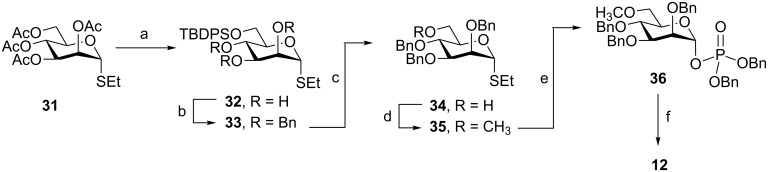
Reagents and conditions: (a) (i) NaOCH_3_, CH_3_OH; (ii) TBDPSCl, imidazole, DMF, 78%; (b) BnBr, NaH, TBAI, 84%; (c) TBAF, THF, 83%; (d) CH_3_I, NaH, DMF, 87%; (e) HO-P(O)(OBn)_2_, NIS, AgOTf, CH_2_Cl_2_, 70%; (f) H_2_, Pd(OH)_2_–C, NaHCO_3_, CH_3_OH, 91%.

**Scheme 5:**
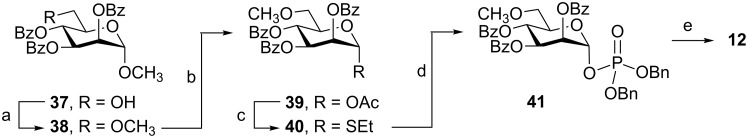
Reagents and conditions: (a) Ag_2_O, CaSO_4_, CH_3_I, 52%; (b) Ac_2_O–HOAc–H_2_SO_4_, 70:30:1, 96%; (c) EtSH, BF_3_·OEt_2_, CH_2_Cl_2_, 75%; (d) HO-P(O)(OBn)_2_, NIS, AgOTf, CH_2_Cl_2_, 89%; (e) (i) H_2_, Pd(OH)_2_–C, toluene, Et_3_N, pyridine; (ii) CH_3_OH–H_2_O–Et_3_N, 5:2:1, 85%.

Two routes, differing in the choice of protecting groups, were explored to produce the 6-methoxy Man*p*-1P derivative **12** (Scheme 4 and Scheme 5). In one route, the C-2, C-3, and C-4 hydroxy groups of the mannose residues were protected with benzyl ethers and in the other they were protected with benzoyl esters. The overall yields of these two methods were 30% and 17%, respectively. In the first method (Scheme 4), the initial step was the conversion, in 78% yield, of the fully acetylated thioglycoside **31** [28] into silyl ether **32** by treatment with sodium methoxide and then *tert*-butyldiphenylchlorosilane in DMF. Benzylation of **32** using benzyl bromide and sodium hydride gave **33** in 84% yield. The TBDPS group was then cleaved and replaced with a methyl group to give the 6-methoxy compound **35** in 72% yield over two steps. The protected dibenzyl phosphate **36** was formed in 70% yield by phosphorylation as described for the synthesis of **9**–**11**. Catalytic hydrogenolysis in the presence of NaHCO_3_ was used to cleave all the benzyl groups, which gave the 6-methoxy Man*p*-1P derivative **12** in 91% yield.

The second route to **12** began with methyl 2,3,4-tri-*O*-benzoyl-α-D-mannopyranoside (**37**) [29] and is illustrated in Scheme 5. Methylation of the free OH, even under mildly basic conditions (e.g., Ag_2_O–CaSO_4_), led to significant amounts of acyl group migration, and the desired product was obtained in only 52% yield. Nevertheless, enough material was produced to move forward. Acetolysis conditions were used to replace the methyl group at the anomeric center in **38** with an acetyl group, resulting in a 96% yield of **39**. Thioglycosylation, followed by coupling of the resulting thioglycoside donor **40** (obtained in 75% yield) with dibenzyl phosphate, gave phosphate **41** in a yield of 67% over the two steps. The 6-methoxy Man*p*-1P analogue **12** was obtained by catalytic hydrogenolysis of the benzyl ethers followed by treatment with CH_3_OH–H_2_O–Et_3_N 5:2:1 providing **12** in 85% yield over two steps.

### Synthesis of 6-deoxy derivative **13**

**Scheme 6:**
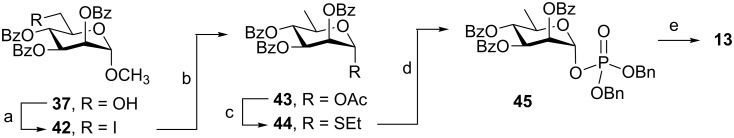
Reagents and conditions: (a) PPh_3_, imidazole, I_2_, 65%; (b) (i) Ac_2_O–HOAc–H_2_SO_4_, 35:15:1; (ii) Pd–C, H_2_, Et_3_N, EtOAc, 72%; (c) EtSH, BF_3_·OEt_2_, CH_2_Cl_2_, 89%, α/β 4:1; (d) HO-P(O)(OBn)_2_, NIS, AgOTf, CH_2_Cl_2_, 67%; (e) (i) H_2_, Pd(OH)_2_–C, toluene, Et_3_N, pyridine; (ii) CH_3_OH–H_2_O–Et_3_N, 5:2:1, 72%.

The synthesis of the 6-deoxy Man*p*-1P analogue **13** used an intermediate (**37**) prepared in the course of the synthesis of the 6-methoxy analogue (Scheme 6). First, the hydroxy group of **37** was converted to the corresponding iodide in 65% yield, by using triphenylphospine and iodine. The product, **42**, was then subjected to acetolysis and catalytic hydrogenation, which gave 6-deoxy glycosyl acetate derivative **43** in 72% yield. The subsequent thioglycosylation, phosphorylation and deprotection steps proceeded, as outlined above, to give the 6-deoxy Man*p*-1P **13** in 43% yield over four steps.

### Evaluation of **9**–**13** as substrates for GDP-Man pyrophosphorylase

**Figure 3:**
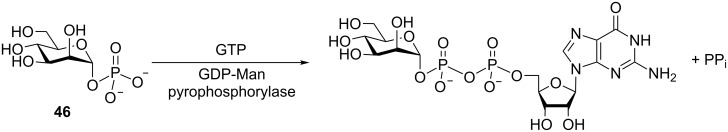
Reaction catalyzed by GDP-ManPP.

With **9**–**13** in hand, each was evaluated as a substrate for the *S. enterica* GDP-ManPP. Before doing that, the recombinant protein was produced and the natural substrate for the enzyme, Man*p*-1P (**46**, Figure 3), was evaluated by incubation with the enzyme and GTP. The reaction was monitored by HPLC (Figure S1 in Supporting Information File 1) and stopped when the complete consumption of GTP was observed. Simultaneous with the loss of the GTP was the appearance of the signal for a new product, which was found to elute at a retention time similar to that for an authentic sample of GDP-Man*p.* The product was isolated, and analysis by high-resolution electrospray ionization mass spectrometry revealed an ion with *m*/*z* = 604.0691, which corresponds to the [M − H]^−^ ion (calcd *m*/*z* = 604.0699) of GDP-Man*p*.

**Figure 4:**
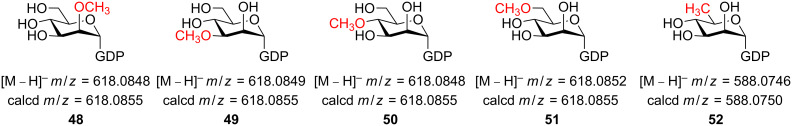
Structure of modified GDP-Man derivatives **48**–**52** produced from **9**–**13**.

Having established that the enzyme GDP-ManPP was active, we carried out the same incubations for **9**–**13**, and in all cases the corresponding GDP-Man*p* analogue peaks could be observed (Figure S2 in Supporting Information File 1). However, in the case of **11** and **9**, a peak corresponding to GDP, resulting from hydrolysis of the GDP-sugar, was also observed, and, in the case of **9**, a much smaller amount of the GDP-Man*p* analogue was produced. To confirm the identity of each GDP-Man*p* analogue, the product peaks were isolated and analysed by electrospray ionization mass spectrometry. For the reactions involving **9**–**12** a signal at *m*/*z ≈* 618 was observed, as would be expected for the [M − H]^−^ ion of the methylated GDP-Man derivatives (**48**–**51**, Figure 4). Similarly, for the reaction with **13**, a signal at *m*/*z* ≈ 588 was observed in the mass spectrum consistent with the 6-deoxy GDP-Man derivative **52**.

### Relative activity of Man*p*-1P analogues with GDP-ManPP

**Figure 5:**
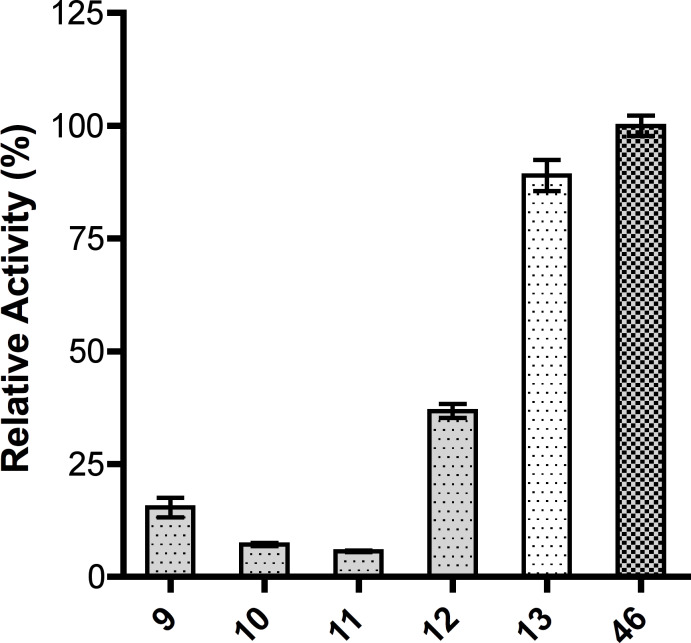
Comparison of the relative activity of synthetic Man*p*-1P analogues **9**–**13** for GDP-ManPP, with that of the parent compound **46**. Error bars represent the standard deviation of duplicate reactions.

**Figure 6:**
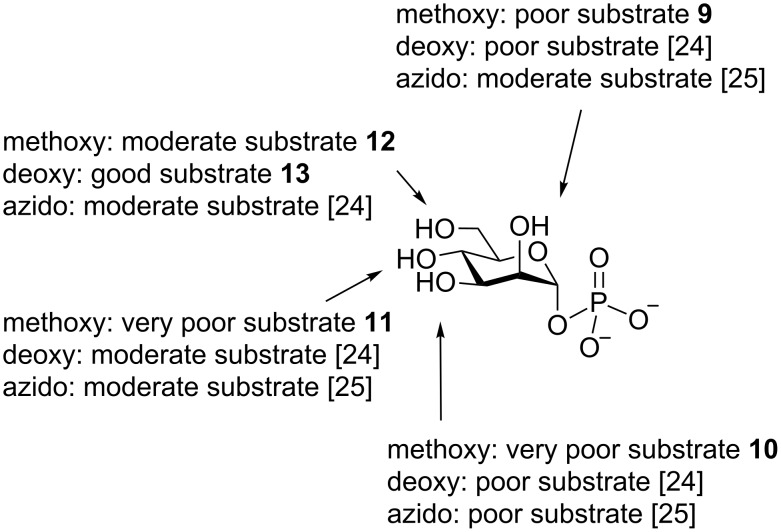
Summary of the substrate specificity of GDP-ManPP. Data from previous studies on the enzyme are also included as indicated [24–25].

After it was established that all five Man*p*-1P analogues could serve as substrates for GDP-ManPP, the relative activity with each was assessed. This was done by using an established colorimetric activity assay, which relies on the detection of the pyrophosphate (PPi, Figure 3) formed as a byproduct of the enzymatic reaction [30]. As illustrated in Figure 5, all five synthetic derivatives **9**–**13** were active as substrates, although at lower levels than the parent compound **46**. The 6-methoxy (**12**) and 6-deoxy (**13**) analogues, demonstrated moderate to good relative activities, while the 2-methoxy (**9**), 3-methoxy (**10**), and 4-methoxy (**11**) compounds showed much lower activities. For example, the 2-methoxy, 3-methoxy, and 4-methoxy analogues displayed a 6-, 14-, and 17-fold decrease relative to **46**, respectively. Because both the 6-deoxy and 6-methoxy analogues (**12** and **13**) showed relatively good activity it is likely that this hydroxy group does not interact significantly with the enzyme. On the other hand, because the 2-methoxy, 3-methoxy, and 4-methoxy compounds all showed a large decrease in activity, it is likely that these positions are bound tightly in the active site of the enzyme. A graphical summary of the substrate specificity for GDP-ManPP is shown in Figure 6.

### Kinetic analysis of Man*p*-1P analogues with GDP-ManPP

To better understand how these **9**–**13** interact with GDP-ManPP, kinetic analyses were performed by using the colorimetric activity assay mentioned above (Table 1). Both the 6-methoxy Man*p*-1P (**12**) and 6-deoxy Man*p*-1P (**13**) derivatives bind relatively well to the enzyme, showing only a two- or three-fold increase in *K*_M_, respectively, compared to the native Man*p*-1P donor **46**. The turnover rate of 6-methoxy analogue **12** is, however, much lower than the 6-deoxy counterpart (**13**) and the natural substrate **46**, as substantiated by a greater than 10-fold decrease in *k*_cat_. Taken together, these results suggest that the C-6 hydroxy group does not engage in any critical hydrogen-bonding interactions and that a bulky substituent interferes with the rate of substrate turnover. The binding of the 2-methoxy (**9**) and 4-methoxy (**11**) analogues is very weak compared to the native substrate, as seen by the greater then 100-fold increase in *K*_M_; consequently, the turnover rates are also low. The binding between 3-methoxy analogue **10** is moderate, with only a five-fold increase in the observed *K*_M_, but it shows an extremely low turnover rate. These results all suggest that GDP-ManPP is not tolerant of bulky substituents at the C-2, C-3, and C-4 positions, which is consistent with the results obtained from their relative activity. It should be noted that these trends are consistent with earlier studies of the enzyme using deoxygenated or azido analogues [24–25].

**Table 1:** *K*_M_, *k*_cat_, and *k*_cat_/*K*_M_ of GDP-ManPP kinetic studies.

compound	*K*_M_ (μM)	*k*_cat_ (min^−1^)	*k*_cat_/*K*_M_ (min^−1^·μM^−1^)

**9** (2-methoxy analogue)	4000 ± 1100	70 ± 11	(2 ± 1) × 10^−2^
**10** (3-methoxy analogue)	200 ± 72	5.2 ± 0.7	(2.6 ± 0.1) × 10^−2^
**11** (4-methoxy analogue)	3400 ± 870	31 ± 4.7	(9 ± 5) × 10^−3^
**12** (6-methoxy analogue)	120 ± 18	27 ± 1	0.23 ± 0.06
**13** (6-deoxy analogue)	70 ± 13	300 ± 13	4 ± 1
**46** (Man-1P)	40 ± 6	360 ± 16	9 ± 3

## Conclusion

In this paper, we report the synthesis of a panel of methoxy and deoxy analogues of Man*p*-1P. Five analogues, **9**–**13**, in which one of the hydroxy groups was methylated or deoxygenated were generated by chemical synthesis, and the ability of these compounds to be converted to the corresponding GDP-Man*p* analogues by GDP-ManPP from *S. enterica* was evaluated. All the derivatives acted as substrates for GDP-ManPP, but with uniformly lower activity than the natural substrate Man-1P. The results suggest that the C-2, C-3, and C-4 hydroxy groups of Man*p*-1P are bound within the active site of GDP-ManPP and the addition of a methyl group at these positions is tolerated very poorly. Conversely, the addition of a methyl group to, or deoxygenation of, O-6 had a much smaller effect, suggesting that this position protrudes from the active site, or is accommodated in a pocket that can tolerate either of these modifications. These results are consistent with earlier studies of this enzyme, which were focused on deoxygenated and azido derivatives [24–25]. Considered together, our studies and those published previously suggest that this enzyme can be used to access deoxy and azido derivatives of GDP-Man on a preparative scale, but that the synthesis of analogues containing more sterically demanding groups is likely to be only possible when the modifications are present on O-6.

## Experimental

Detailed experimental procedures can be found in Supporting Information File 1.

## Supporting Information

File 1 Detailed experimental procedures.
