# Supplementary material for: Studies on the substrate specificity of a GDP-mannose pyrophosphorylase from Salmonella enterica
Source: Beilstein J Org Chem. 2012 Aug 1;8:1219–26. doi: 10.3762/bjoc.8.136 (PMC3458741; doi:10.3762/bjoc.8.136)
Supplement: File 1 — Detailed experimental procedures. [file Beilstein_J_Org_Chem-08-1219-s001.pdf]

# Supporting Information

for

# Studies on the substrate specificity of a GDP-mannose pyrophosphorylase from *Salmonella enterica*

Lu Zou, Ruixiang Blake Zheng and Todd L. Lowary*

Address: Alberta Glycomics Centre and Department of Chemistry, The University of Alberta, Edmonton, AB T6G 2G2, Canada

Email: Todd L. Lowary* - tlowary@ualberta.ca

* Corresponding author

## Detailed experimental procedures

General methods ........................................ S2

Synthesis procedures of **9**–**45** ........................................ S3

Enzymatic synthesis of GDP-Man*p* analogues ........................................ S30

Characterization of GDP-Man*p* analogues ........................................ S31

GDP-Man*p* and GDP-Man*p* analogues HPLC traces ........................................ S32

GDP-ManPP activity assay ........................................ S33

References ........................................ S35

## General methods

All reagents used were purchased from commercial sources and were used without further purification unless noted. The solvents used were purified by successive passage through columns of alumina and copper under an argon atmosphere. All reactions were carried out under a positive pressure of argon at room temperature unless indicated otherwise. The reactions were monitored by analytical TLC on silica get 60-$F_{254}$ (0.25 mm, Silicycle, Quebec, Canada), and the spots were visualized under UV light (254 nm) or stained by charring with acidified anisaldehyde solution in ethanol. Organic solvents were evaporated under reduced pressure and the products were purified by column chromatography on silica gel (230–400 mesh, Silicycle, Quebec, Canada) or SepPak $C_{18}$ reverse phase cartridges (Waters). Before use, the cartridges were prewashed with 10 mL of MeOH followed by 20 mL of $H_2O$. Optical rotations were measured on Perkin Elmer 241 polarimeter at ambient temperature in units of degree·mL/(g·dm). $^{1}$H NMR spectra were recorded at 400, 500 or 600 MHz and chemical shifts were referenced to $CDCl_3$ (7.26 ppm), $CD_3OD$ (3.31 ppm), or $D_2O$ (4.79 ppm). $^{13}$C NMR spectra were recorded at 100 or 125 MHz and chemical shifts were referenced to $CDCl_3$ (77.1 ppm) or $CD_3OD$ (49.0 ppm). $^{31}$P NMR spectra were recorded at 162 or 202 MHz and chemical shifts were referenced to external $H_3PO_4$ (0.0 ppm). Assignments of NMR spectra were based on two-dimensional experiments ($^{1}$H,$^{1}$H COSY or HSQC). The stereochemistry of the anomeric centres of the pyranose rings was confirmed by measuring the $^{1}J_{C\text{-}1,H\text{-}1}$ coupling constant of coupled HSQC spectra [1]. Electrospray mass spectra were recorded on an Agilent Technologies 6220 TOF.

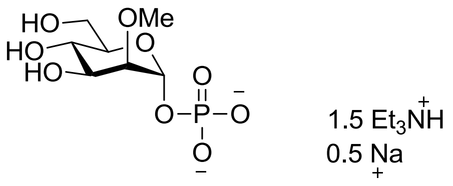

**2-*O*-Methyl-α-D-mannopyranosyl phosphate mixed-triethylammonium-sodium salt (9)**

The protected phosphate **18** (118 mg, 0.2 mmol) was dissolved in a mixture of toluene–$Et_3N$–pyridine (15:2:3, 2 mL). To this solution was added 20% $Pd(OH)_2$–C (11 mg) and the reaction mixture was stirred for 2 days under a hydrogen atmosphere. The catalyst was then removed by filtration through Celite and the filtrate was concentrated to afford a pale yellow syrup. Without further purification, this crude compound was dissolved in MeOH–$H_2O$–$Et_3N$ (5:2:1, 4 mL) and the mixture was stirred for 2 days. The organic impurities were removed using a SepPak $C_{18}$ cartridge eluting with $H_2O$. The $H_2O$ fraction was then lyophilized to afford **9** (80.2 mg, 92%) as a white solid. $[\alpha]_D$ = +24.8 (*c* 0.3, $H_2O$); $^1$H NMR (600 MHz, $D_2O$) δ 5.53 (dd, *J* = 8.1, 1.7 Hz, 1H, H-1), 3.93 (dd, *J* = 9.9, 3.5 Hz, 1H, H-3), 3.86 (dd, *J* = 12.2, 2.2 Hz, 1H, H-6a), 3.80 (ddd, *J* = 9.9, 6.0, 2.0 Hz, 1H, H-5), 3.71 (dd, *J* = 12.2, 6.0 Hz, 1H, H-6b), 3.61 (dd, *J* = 3.5, 1.9 Hz, 1H, H-2), 3.56 (app t, *J* = 10.0 Hz, 1H, H-4), 3.48 (s, 3H, $OCH_3$), 3.19 (q, *J* = 7.3 Hz, 9H, 1.5 × $N(CH_2CH_3)_3$), 1.26 (t, *J* = 7.3 Hz, 13.5H, 1.5 × $N(CH_2CH_3)_3$). $^{13}$C NMR (126 MHz, $D_2O$) δ 93.4 (d, $^2J_{C,P}$ = 5.2 Hz, 1C, C-1, $^1J_{C,H}$ = 177.9 Hz), 81.6 (d, $^3J_{C,P}$ = 7.9 Hz, 1C, C-2), 74.3 (C-5), 70.7 (C-3), 67.9 (C-4), 61.9 (C-6), 59.8 ($OCH_3$), 47.7 ($N(CH_2CH_3)_3$), 9.2 ($N(CH_2CH_3)_3$). $^{31}$P NMR (162 MHz, $CD_3OD$) δ 0.60. HRMS (ESI) *m/z* Calcd for $C_7H_{14}O_9P$ $[M - H]^-$: 273.0381. Found: 273.0381.

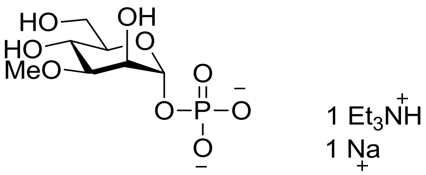


**3-*O*-Methyl-α-D-mannopyranosyl phosphate mixed-triethylammonium-sodium salt (10)**

The protected phosphate **23** (33 mg, 0.06 mmol) was dissolved in a mixture of toluene–$Et_3N$–pyridine (15:2:3, 2.8 mL). To this solution was added 20% $Pd(OH)_2$–C (4 mg). The reaction mixture was stirred for 4 days under an atmosphere of hydrogen. The catalyst was then removed by filtration through Celite and the filtrate was concentrated to afford a pale yellow syrup. The syrup was dissolved in MeOH–$H_2O$–$Et_3N$ (5:2:1, 4 mL) and the mixture was stirred for 3 days. Any organic impurities were removed using a SepPak $C_{18}$ cartridge eluting with $H_2O$ and the filtrate was lyophilized to afford **10** (16 mg, 67%) as a white solid. $[\alpha]_D$ = +16.5 (*c* 0.1, $H_2O$); $^1$H NMR (500 MHz, $D_2O$) δ 5.42 (d, *J* = 7.8 Hz, 1H, H-1), 4.21 (br. s, 1H, H-2), 3.87-3.82 (m, 2H, H-5, H-6a), 3.74 (dd, *J* = 11.8, 5.0 Hz, 1H, H-6b), 3.69 (app t, *J* = 9.8 Hz, 1H, H-4), 3.59 (dd, *J* = 9.6, 2.0 Hz, 1H, H-3), 3.44 (s, 3H, $OCH_3$), 3.18 (q, *J* = 7.3 Hz, 6H, $N(CH_2CH_3)_3$), 1.26 (t, *J* = 7.2 Hz, 9H, $N(CH_2CH_3)_3$). $^{13}$C NMR (126 MHz, $D_2O$) δ 96.8 (d, $^2J_{C,P}$ = 5.2 Hz, 1C, C-1, $^1J_{C,H}$ = 178.5 Hz), 80.27 (C-3), 74.4 (C-5), 67.3 (d, $^3J_{C,P}$ = 8.2 Hz, 1C, C-2), 66.5 (C-4), 61.8 (C-6), 57.2 ($OCH_3$), 47.7 ($N(CH_2CH_3)_3$), 9.2 ($N(CH_2CH_3)_3$). $^{31}$P NMR (162 MHz, $D_2O$) δ −1.82. HRMS (ESI) *m/z* Calcd for $C_7H_{14}O_9P$ $[M - H]^-$: 273.0381. Found: 273.0371.

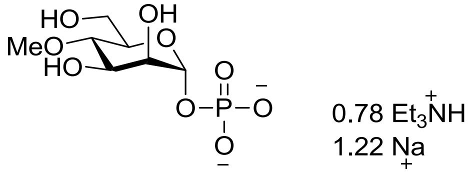


**4-*O*-Methyl-α-D-mannopyranosyl phosphate mixed-triethylammonium-sodium salt (11)**

The protected phosphate **30** (220 mg, 0.38 mmol) was dissolved in a mixture of toluene–$Et_3N$–pyridine (15:2:3, 4 mL). To the solution was added $Pd(OH)_2$–C (40 mg). The reaction mixture was stirred for 3 days under a hydrogen atmosphere. Then the catalyst was removed by filtration through Celite and the filtrate was concentrated to afford a pale yellow syrup. Without purification the crude compound was dissolved in MeOH–$H_2O$–$Et_3N$ (5:2:1, 8 mL) and the mixture was stirred for 2 days. Organic impurities were removed using a SepPak $C_{18}$ cartridge eluting with $H_2O$. The $H_2O$ fraction was lyophilized to yield **11** (101 mg, 70%) as a white solid. $[\alpha]_D$ = +28.8 (*c* 0.3, $H_2O$); $^1$H NMR (500 MHz, $D_2O$) δ 5.33 (d, *J* = 8.1 Hz, 1H, H-1), 3.97 (dd, *J* = 9.6, 3.2 Hz, 1H, H-3), 3.94 (br. s, 1H, H-2), 3.85 (d, *J* = 12.0 Hz, 1H, H-6a), 3.83–3.78 (m, 1H, H-5), 3.73 (dd, *J* = 12.0, 5.2 Hz, 1H, H-6b), 3.53 (s, 3H, OC*H*$_3$), 3.42 (app t, *J* = 9.8 Hz, 1H, H-4), 3.18 (q, *J* = 7.3 Hz, 4.7H, 0.78 × N(C*H*$_2$CH$_3$)$_3$), 1.26 (t, *J* = 7.3 Hz, 7H, 0.78 × N(CH$_2$C*H*$_3$)$_3$). $^{13}$C NMR (126 MHz, $D_2O$) δ 96.5 (d, $^2J_{C,P}$ = 5.2 Hz, 1C, C-1, $^1J_{C,H}$ = 177.3 Hz), 77.7 (C-4), 73.2 (C-5), 72.0 (d, $^3J_{C,P}$ = 8.0 Hz, 1C, C-2), 70.8 (C-3), 61.7 (C-6), 61.1 (O*C*H$_3$), 47.7 (N(*C*H$_2$CH$_3$)$_3$), 9.2 (N(CH$_2$*C*H$_3$)$_3$). $^{31}$P NMR (162 MHz, $D_2O$) δ −0.29. HRMS (ESI) *m/z* Calcd for $C_7H_{14}O_9P$ $[M - H]^-$: 273.0381. Found: 273.0377.

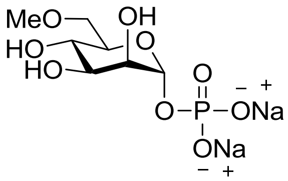


**6-*O*-Methyl-α-D-mannopyranosyl phosphate disodium salt (12)**

The protected α-D-mannopyranosyl phosphate **36** (181 mg, 0.25 mmol) was dissolved in MeOH (7.5 mL). A 1 M $NaHCO_3$ solution (0.75 mL) and 20% $Pd(OH)_2$–C (80 mg) were added to the mixture. The mixture was stirred for 6 days under a hydrogen atmosphere. Then the catalyst was removed by filtration through Celite and the filtrate was concentrated. Organic impurities were removed using a SepPak $C_{18}$ cartridge eluting with $H_2O$. The filtrate was lyophilized to afford **12** (72 mg, 91%) as a white amorphous solid $[\alpha]_D$ = +30.3 (*c* 1.1, $H_2O$); $^1H$ NMR (400 MHz, $D_2O$) δ 5.30 (dd, *J* = 8.6, 1.1 Hz, 1H, H-1), 3.99–3.91 (m, 3H, H-2, H-3, H-5), 3.75 (dd, *J* = 10.9, 2.2 Hz, 1H, H-6a), 3.64 (dd, *J* = 10.9, 6.3 Hz, 1H, H-6b), 3.59 (dd, *J* = 10.0, 9.4 Hz, 1H, H-4), 3.40 (s, 3H, $OCH_3$). $^{13}C$ NMR (100 MHz, $CDCl_3$) δ 95.4 (d, $^2J_{C,P}$ = 4.1 Hz, 1C, C-1, $^1J_{C,H}$ = 170.9 Hz), 72.1 (C-6), 71.9 (C-5), 71.5 (d, $^3J_{C,P}$ = 6.4 Hz, 1C, C-2), 70.5 (C-3), 67.5 (C-4), 56.0 ($OCH_3$). $^{31}P$ NMR (162 MHz, $D_2O$) δ 2.00. HRMS (ESI) *m/z* Calcd for $C_7H_{14}O_9P$ $[M - H]^-$: 273.0381. Found: 273.0380.

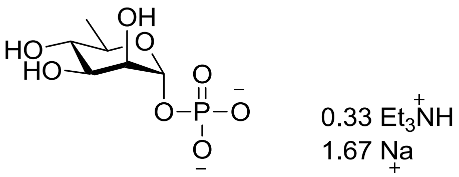


**6-Deoxy-α-D-mannopyranosyl phosphate mixed-triethylammonium-sodium salt (13)**

The protected phosphate **45** (59 mg, 0.08 mmol) was dissolved in a mixture of toluene–$Et_3N$–pyridine (15:2:3, 2.8 mL). To the solution was added 20% $Pd(OH)_2$–C (12 mg). The reaction mixture was stirred for 2 days under a hydrogen atmosphere. The catalyst was then removed by filtration through Celite and the filtrate was concentrated to afford pale yellow syrup. Without further purification, the crude compound was dissolved in MeOH–$H_2O$–$Et_3N$ (5:2:1, 8 mL) and the mixture was stirred for 6 days. The organic impurities were removed using a SepPak $C_{18}$ cartridge eluting with $H_2O$. The $H_2O$ fraction was lyophilized to afford **13** (18 mg, 72%) as a white solid $[\alpha]_D$ = +18.1 (*c* 0.2, $H_2O$); $^1$H NMR (500 MHz, $D_2O$) $\delta$ 5.38 (d, *J* = 7.8 Hz, 1H, H-1), 4.04 (br. s, 1H, H-2), 4.01–3.92 (m, 2H, H-5, H-3), 3.50 (app t, *J* = 9.8 Hz, 1H, H-4), 3.27 (q, *J* = 7.3 Hz, 2H, 0.33 × $N(CH_2CH_3)_3$ [$N(C\mathit{H}_2CH_3)_3$]), 1.38–1.32 (m, 6H, H-6, 0.33 × $N(CH_2C\mathit{H}_3)_3$). $^{13}$C NMR (126 MHz, $D_2O$) $\delta$ 96.4 (d, $^2J_{C,P}$ = 5.4 Hz, 1C, C-1, $^1J_{C,H}$ = 171.2 Hz), 73.2 (C-4), 71.8 (d, $^3J_{C,P}$ = 8.3 Hz, 1C, C-2), 70.7 (C-3), 70.1 (C-5), 47.7 ($N(\mathit{C}H_2CH_3)_3$), 17.8 (C-6), 9.2 ($N(CH_2\mathit{C}H_3)_3$). $^{31}$P NMR (162 MHz, $D_2O$) $\delta$ −0.71. HRMS (ESI) *m/z* Calcd for $C_6H_{12}O_8P$ $[M - H]^-$: 243.0275. Found: 243.0275.

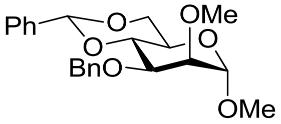

**Methyl 3-*O*-benzyl-4,6-*O*-benzylidene-2-*O*-methyl-α-D-mannopyranoside (15)**

Methyl 3-*O*-benzyl-4,6-*O*-benzylidene-α-D-mannopyranoside **14** [2] (1.43 g, 3.8 mmol) was dissolved in DMF (15 mL) and the solution was cooled to 0 °C. After NaH (60% NaH in mineral oil, 274 mg, 6.9 mmol) was added, the mixture was stirred for 10 min before the addition of $CH_3I$ (0.36 mL, 5.76 mmol). The reaction mixture was stirred overnight and then quenched by the addition of MeOH (1 mL). The solution was diluted with $CH_2Cl_2$, washed with distilled water and brine, and the organic layer was dried over $Na_2SO_4$. Evaporation of the solvent and chromatography (hexane–EtOAc 5:1) of the resulting residue gave **15** (1.17 g, 80%) as a colorless syrup. $[\alpha]_D$ = +55.6 (*c* 0.5, $CHCl_3$); $^1$H NMR (400 MHz, $CDCl_3$) δ 7.60–7.26 (m, 10H, ArH), 5.67 (s, 1H, $O_2CH$Ph), 4.92 (d, *J* = 12.3 Hz, 1H, $CH_2$Ph), 4.80 (br. s, 1H, H-1), 4.77 (d, *J* = 12.3 Hz, 1H, $CH_2$Ph), 4.31 (dd, *J* = 10.0, 4.3 Hz, 1H, H-6a), 4.22 (app t, *J* = 9.5 Hz, 1H, H-4), 4.00 (dd, *J* = 9.9, 2.7 Hz, 1H, H-3), 3.92 (app t, *J* = 10.5 Hz, 1H, H-6b), 3.85–3.81 (m, 1H, H-5), 3.65–3.64 (m, 1H, H-2), 3.61 (s, 1H, $OCH_3$), 3.39 (s, 1H, $OCH_3$). $^{13}$C NMR (101 MHz, $CDCl_3$) δ 138.7 (Ar), 137.7 (Ar), 128.8 (Ar), 128.3 (Ar), 128.2 (Ar), 127.6 (Ar), 127.5 (Ar), 126.1 (Ar), 101.5 ($O_2C$HPh), 100.1 (C-1), 79.5 (C-2), 79.2 (C-4), 76.2 (C-3), 73.1 ($CH_2$Ph), 68.9 (C-6), 64.2 (C-5), 60.1 ($OCH_3$), 54.9 ($OCH_3$). HRMS (ESI) *m/z* Calcd for $C_{22}H_{26}O_6Na$ $[M + Na]^+$: 409.1622. Found: 409.1616.

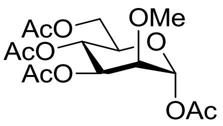


**1,3,4,6-Tetra-*O*-acetyl-2-*O*-methyl-α-D-mannopyranose (16)**

Monosaccharide **15** (1.17 g, 3 mmol) was dissolved in $Ac_2O$–HOAc–$H_2SO_4$ (35:15:1 v/v/v, 18.36 mL). After being stirred overnight the mixture was diluted with $CH_2Cl_2$, washed with distilled water, $NaHCO_3$ (satd aq soln) and brine. The organic layer was dried over $Na_2SO_4$ and concentrated, and the crude residue purified by chromatography (hexane–EtOAc 3:2) to afford **16** (894 mg, 81%) as a pale yellow syrup. $[\alpha]_D$ = +56.2 (*c* 0.5, $CHCl_3$); $^1$H NMR (400 MHz, $CDCl_3$) δ 6.04 (d, *J* = 1.9 Hz, 1H, H-1), 5.23 (app t, *J* = 10.1 Hz, 1H, H-4), 5.08 (dd, *J* = 10.1, 3.3 Hz, 1H, H-3), 4.12 (dd, *J* = 12.4, 4.7 Hz, 1H, H-6a), 3.92 (dd, *J* = 12.4, 2.3 Hz, 1H, H-6b), 3.87 (ddd, *J* = 10.0, 4.6, 2.3 Hz, 1H, H-5), 3.50 (dd, *J* = 3.2, 2.1 Hz, 1H, H-2), 3.36 (s, 3H, $OCH_3$), 2.01 (s, 3H, $C(O)CH_3$), 1.95 (s, 3H, $C(O)CH_3$), 1.93 (s, 3H, $C(O)CH_3$), 1.90 (s, 3H, $C(O)CH_3$). $^{13}$C NMR (101 MHz, $CDCl_3$) δ 90.5 (C-1), 76.8 (C-2), 70.7 (C-3, C-5), 65.6 (C-4), 62.0 (C-6), 59.3 ($OCH_3$), 20.7 ($C(O)CH_3$), 20.6 ($C(O)CH_3$), 20.5 ($C(O)CH_3$), 20.4 ($C(O)CH_3$). HRMS (ESI) *m/z* Calcd for $C_{15}H_{22}O_{10}Na$ $[M + Na]^+$: 385.1105. Found: 385.1099.

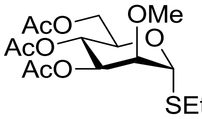


**Ethyl 3,4,6-tri-*O*-acetyl-2-*O*-methyl-1-thio-α-D-mannopyranoside (17)**

Tetraacetate **16** (888 mg, 2.45 mmol) and ethanethiol (272 µL, 3.68 mmol) were dissolved in $CH_2Cl_2$ (12 mL) and cooled to 0 °C before $BF_3{\cdot}OEt_2$ (615 µL, 4.9 mmol) was added. The reaction mixture was stirred for 18 h and then diluted with $CH_2Cl_2$, and washed with $NaHCO_3$ (satd aq soln), distilled water and brine. The organic layer was dried over $Na_2SO_4$ and concentrated, and the crude residue was purified by chromatography (hexane–EtOAc 3:1 → 2:1)

to give **17** (583 mg, 65%) a pale yellow syrup. $[\alpha]_D$ = +85.7 (*c* 0.5, $CHCl_3$); $^1$H NMR (400 MHz, $CDCl_3$) δ 5.34 (s, 1H, H-1), 5.29–5.18 (m, 1H, H-4), 5.07 (dd, *J* = 9.8, 3.1 Hz, 1H, H-3), 4.29–4.14 (m, 2H, H-5, H-6a), 3.98 (d, *J* = 10.3 Hz, 1H, H-6b), 3.65 (d, *J* = 1.2 Hz, 1H, H-2), 3.37 (s, 3H, OC$H_3$), 2.69–2.45 (m, 2H, SC$H_2$CH$_3$), 1.97 (d, *J* = 13.8 Hz, 9H, C(O)C$H_3$ × 3), 1.24 (t, *J* = 7.4 Hz, 3H, SCH$_2$C$H_3$). $^{13}$C NMR (101 MHz, $CDCl_3$) δ 81.3 (C-1), 79.3 (C-2), 71.6 (C-3), 68.8 (C-5), 66.5 (C-4), 62.4 (C-6), 58.6 (O*C*H$_3$), 25.3 (S*C*H$_2$CH$_3$), 20.7 (C(O)*C*H$_3$), 20.6 (C(O)*C*H$_3$), 20.6 (C(O)*C*H$_3$), 14.8 (SCH$_2$*C*H$_3$). HRMS (ESI) *m/z* Calcd for $C_{15}H_{24}O_8SNa$ $[M + Na]^+$: 387.1084. Found: 387.1081.

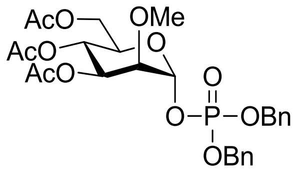


**Dibenzyl 3,4,6-tri-*O*-acetyl-2-*O*-methyl-α-D-mannopyranosyl phosphate (18)**

Thioglycoside **17** (547 mg, 1.5 mmol), dibenzyl phosphate (709 mg, 2.55 mmol), and powdered 4 Å molecular sieves were dissolved in dry $CH_2Cl_2$ (14 mL) and stirred for 40 min. *N*–iodosuccinimide (507 mg, 2.25 mmol) and silver trifluoromethanesulfonate (116 mg, 0.45 mmol) were then added at −30 °C. The reaction mixture was stirred for 40 min from −30 °C to −20 °C, and then filtered through Celite. The filtrate was diluted with $CH_2Cl_2$, and washed with $Na_2S_2O_3$ (satd aq soln), $NaHCO_3$ (satd aq soln), distilled water and brine. The organic layer was dried over $Na_2SO_4$ then concentrated and the crude residue was purified by chromatography (hexane–EtOAc 1:1) to yield **18** (729 mg, 84%) as a colorless syrup. $[\alpha]_D$ = +38.4 (*c* 0.4, $CHCl_3$); $^1$H NMR (600 MHz, $CDCl_3$) δ 7.37–7.31 (m, 10H, ArH), 5.68 (dd, *J* = 6.4, 2.0 Hz, 1H, H-1), 5.31 (app t, *J* = 10.2 Hz, 1H, H-4), 5.18 (dd, *J* = 10.1, 3.2 Hz, 1H, H-3), 5.13–5.03 (m, 4H, C$H_2$Ph), 4.16 (dd, *J* = 12.4, 4.6 Hz, 1H, H-6a), 3.96 (ddd, *J* = 10.1, 4.6, 2.1 Hz, 1H, H-5), 3.90 (dd, *J* =

12.4, 2.3 Hz, 1H, H-6b), 3.50 (dd, $J$ = 3.1, 2.2 Hz, 1H, H-2), 3.37 (s, 3H, $OCH_3$), 2.06 (s, 3H, $C(O)CH_3$), 2.00 (s, 3H, $C(O)CH_3$), 1.96 (s, 3H, $C(O)CH_3$). $^{13}$C NMR (151 MHz, $CDCl_3$) δ 170.6 ($C$=O), 170.0 ($C$=O), 169.4 ($C$=O), 135.4(1) (d, $^3J_{C,P}$ = 6.6 Hz, 1C, Ar), 135.3(5) (d, $^3J_{C,P}$ = 6.6 Hz, 1C, Ar), 128.8 (Ar), 128.6(9) (Ar), 128.6(8) (Ar), 128.2 (Ar), 128.0 (Ar), 95.2 (d, $^2J_{C,P}$ = 6.0 Hz, 1C, C-1, $^1J_{C,H}$ = 177.7 Hz), 77.5 (d, $^3J_{C,P}$ = 9.5 Hz, 1C, C-2), 70.4(0) (C-5), 70.3(7) (C-3), 69.8 (d, $^2J_{C,P}$ = 5.3 Hz, 1C, $CH_2Ph$), 69.7 (d, $^2J_{C,P}$ = 5.3 Hz, 1C, $CH_2Ph$), 65.5 (C-4), 61.9 (C-6), 59.7 ($OCH_3$), 20.8 ($C(O)CH_3$), 20.6 ($C(O)CH_3$). $^{31}$P NMR (202 MHz, $CDCl_3$) δ −1.80. HRMS (ESI) *m/z* Calcd for $C_{27}H_{33}O_{12}PNa$ $[M + Na]^+$: 603.16019. Found: 603.16003.

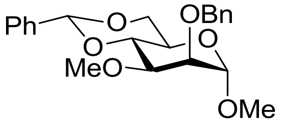


**Methyl 2-*O*-benzyl-4,6-*O*-benzylidene-3-*O*-methyl-α-D-mannopyranoside (20)**

Methyl 2-*O*-benzyl-4,6-*O*-benzylidene-α-D-mannopyranoside [2] **19** (1.29 g, 3.48 mmol) was dissolved in DMF (15 mL) and the solution was cooled to 0 °C. After the addition of NaH (60% NaH in mineral oil, 250 mg, 6.26 mmol), the mixture was stirred for 10 min before the addition of $CH_3I$ (0.33 mL, 5.29 mmol). The reaction mixture was stirred overnight and quenched by the addition of MeOH (1 mL). The solution was then diluted with $CH_2Cl_2$, washed with distilled water and brine, and the organic layer was dried over $Na_2SO_4$. Evaporation of the solvent and chromatography (hexane–EtOAc 8:1) of the residue afforded **20** (1.025 g, 76%) as a colorless syrup. $[\alpha]_D$ = +30.9 ($c$ 2.0, $CHCl_3$); $^1$H NMR (500 MHz, $CDCl_3$) δ 7.54–7.26 (m, 10H, ArH), 5.64 (s, 1H, $O_2CHPh$), 4.84 (d, $J$ = 12.2 Hz, 1H, $CH_2Ph$), 4.75–4.72 (m, 2H, H-1, $CH_2Ph$), 4.28 (dd, $J$ = 10.1, 4.7 Hz, 1H, H-6a), 4.18 (app t, $J$ = 9.6 Hz, 1H, H-4), 3.93–3.86 (m, 2H, H-6b, H-2), 3.83–3.80 (m, 1H, H-5), 3.74 (dd, $J$ = 10.0, 3.2 Hz, 1H, H-3), 3.50 (s, 1H, $OCH_3$), 3.37 (s, 1H, $OCH_3$). $^{13}$C NMR (125 MHz, $CDCl_3$) δ 138.1 (Ar), 137.7 (Ar), 128.9 (Ar), 128.4 (Ar), 128.2

(Ar), 128.0 (Ar), 127.8 (Ar), 126.2 (Ar), 101.7 ($O_2$*C*HPh), 100.3 (C-1), 79.0 (C-4), 78.1 (C-3), 75.6 (C-2), 73.6 (*C*$H_2$Ph), 68.9 (C-6), 64.0 (C-5), 58.8 (O*C*$H_3$), 54.9 (O*C*$H_3$). HRMS (ESI) *m/z* Calcd for $C_{22}H_{26}O_6Na$ $[M + Na]^+$: 409.1622. Found: 409.1613.

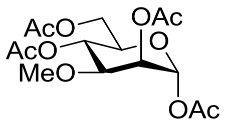


**1,2,4,6-Tetra-*O*-acetyl-3-*O*-methyl-D-mannopyranose (21)**

Monosaccharide **20** (1 g, 2.59 mmol) was dissolved in $Ac_2O$–HOAc–$H_2SO_4$ (35:15:1 v/v/v, 13.26 mL). After being stirred for 2 days, the mixture was diluted with $CH_2Cl_2$ and washed with distilled water, $NaHCO_3$ (satd aq soln) and brine. The organic layer was dried over $Na_2SO_4$ and concentrated, and the crude residue was purified by chromatography (hexane–EtOAc 3:2 → 1:1) to afford **21** (610 mg, 65%) as a pale yellow syrup. $[\alpha]_D$ = +16.7 (*c* 1.5, $CHCl_3$); $^1$H NMR (300 MHz, $CDCl_3$) δ 6.06 (d, *J* = 2.0 Hz, 1H, H-1), 5.30 (dd, *J* = 3.3, 2.1 Hz, 1H, H-2), 5.20 (app t, *J* = 10.0 Hz, 1H, H-4), 4.21 (dd, *J* = 12.4, 5.2 Hz, 1H, H-6a), 4.12–4.06 (m, 1H, H-6b), 3.93 (ddd, *J* = 10.1, 5.2, 2.4 Hz, 1H, H-5), 3.63 (dd, *J* = 9.8, 3.4 Hz, 1H, H-3), 3.34 (s, 3H, OC*H*$_3$), 2.13 (s, 3H, C(O)C*H*$_3$), 2.12 (s, 3H, C(O)C*H*$_3$), 2.06 (s, 3H, C(O)C*H*$_3$), 2.05 (s, 3H, C(O)C*H*$_3$). $^{13}$C NMR (125 MHz, $CDCl_3$) δ 170.7 (*C*=O), 170.0 (*C*=O), 169.7 (*C*=O), 168.1 (*C*=O), 91.0 (C-1, $^1J_{C,H}$ = 177.9 Hz), 76.8 (C-3), 70.8 (C-5), 67.0 (C-4), 66.6 (C-2), 62.4 (C-6), 57.9 (O*C*$H_3$), 20.8(8) (C(O)*C*$H_3$), 20.8(6) (C(O)*C*$H_3$), 20.8(3) (C(O)*C*$H_3$), 20.7(5) (C(O)*C*$H_3$). HRMS (ESI) *m/z* Calcd for $C_{15}H_{22}O_{10}Na$ $[M + Na]^+$: 385.1105. Found: 385.1104.

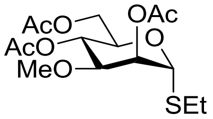


**Ethyl 2,4,6-tri-*O*-acetyl-3-*O*-methyl-1-thio-α-D-mannopyranoside (22)**

Tetraacetate **21** (592 mg, 1.63 mmol) and ethanethiol (182 µL, 2.45 mmol) were dissolved in dry $CH_2Cl_2$ (8 mL) and cooled to 0 °C before the addition of $BF_3{\cdot}OEt_2$ (411 µL, 3.27 mmol). The reaction mixture was stirred overnight and then was diluted with $CH_2Cl_2$ and washed with $NaHCO_3$ (satd aq soln), distilled water and brine. The organic layer was dried over $Na_2SO_4$ and concentrated, and the crude residue was purified by chromatography (hexane–EtOAc 3:1 → 2:1) to afford **22** (307 mg, 52%) as a pale yellow syrup. $[\alpha]_D$ = +71.2 (*c* 1.0, $CHCl_3$); $^1$H NMR (500 MHz, $CDCl_3$) δ 5.40 (dd, *J* = 3.3, 1.8 Hz, 1H, H-2), 5.29 (d, *J* = 2.0 Hz, 1H, H-1), 5.18 (app t, *J* = 9.8 Hz, 1H, H-4), 4.30–4.25 (m, 2H, H-5, H-6a), 4.10–4.07 (m, 1H, H-6b), 3.56 (dd, *J* = 9.8, 3.2 Hz, 1H, H-3), 3.32 (s, 3H, $OCH_3$), 2.69–2.58 (m, 2H, $SCH_2CH_3$), 2.14 (s, 3H, $C(O)CH_3$), 2.08 (s, 3H, $C(O)CH_3$), 2.07 (s, 3H, $C(O)CH_3$), 1.30 (t, *J* = 7.5 Hz, 3H, $SCH_2CH_3$). $^{13}$C NMR (125 MHz, $CDCl_3$) δ 170.7 (*C*=O), 170.3 (*C*=O), 169.8 (*C*=O), 82.6 (C-1), 77.6 (C-3), 69.5 (C-2), 69.1 (C-5), 67.8 (C-4), 62.7 (C-6), 57.8 ($OCH_3$), 25.7 ($SCH_2CH_3$), 21.0 ($C(O)CH_3$), 20.9 ($C(O)CH_3$), 20.8 ($C(O)CH_3$), 14.9 ($SCH_2CH_3$). HRMS (ESI) *m/z* Calcd for $C_{15}H_{24}O_8SNa$ $[M + Na]^+$: 387.1084. Found: 387.1084.

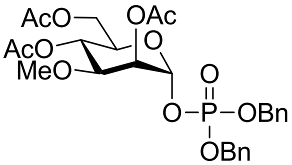


**Dibenzyl 2,4,6-tri-*O*-acetyl-3-*O*-methyl-α-D-mannopyranosyl phosphate (23)**

Thioglycoside **22** (292 mg, 0.8 mmol), dibenzyl phosphate (378 mg, 1.36 mmol), and powdered 4 Å molecular sieves were dissolved in dry $CH_2Cl_2$ (8 mL) and stirred at room temperature for

30 min. *N*–iodosuccinimide (270 mg, 1.2 mmol) and silver trifluoromethanesulfonate (62 mg, 0.24 mmol) were then added at −30 °C. The reaction mixture was stirred for 2 h between −30 °C to −20 °C, and then was filtered through Celite and diluted with $CH_2Cl_2$. The resulting solution was washed with $Na_2S_2O_3$ (satd aq soln), $NaHCO_3$ (satd aq soln), distilled water and brine. The organic layer was dried over $Na_2SO_4$ and concentrated, and the crude residue was purified by chromatography (hexane–EtOAc 1:1) to give **23** (346 mg, 75%) as a colorless syrup. $[\alpha]_D$ = +21.0 (*c* 1.1, $CHCl_3$); $^1$H NMR (500 MHz, $CDCl_3$) δ 7.35–7.26 (m, 10H, ArH), 5.59 (dd, *J* = 6.5, 2.0 Hz, 1H, H-1), 5.21 (dd, *J* = 3.3, 2.3 Hz, 1H, H-2), 5.13 (app t, *J* = 9.9 Hz, 1H, H-4), 5.11–5.02 (m, 4H, $CH_2$Ph), 4.13 (dd, *J* = 12.5, 5.0 Hz, 1H, H-6a), 3.97–3.94 (m, 1H, H-5), 3.92 (dd, *J* = 12.5, 2.5 Hz, 1H, H-6b), 3.50 (dd, *J* = 10.0, 3.5 Hz, 1H, H-3), 3.24 (s, 3H, $OCH_3$), 2.08 (s, 3H, C(O)$CH_3$), 2.03 (s, 3H, C(O)$CH_3$), 1.94 (s, 3H, C(O)$CH_3$). $^{13}$C NMR (151 MHz, $CDCl_3$) δ 170.6 (*C*=O), 169.7 (*C*=O), 169.6 (*C*=O), 135.4 (Ar), 135.3 (Ar), 128.8 (Ar), 128.8 (Ar), 128.7 (Ar), 128.2 (Ar), 128.1 (Ar), 95.6 (d, $^2J_{C,P}$ = 5.7 Hz, 1C, C-1, $^1J_{C,H}$ = 183.1 Hz), 76.1 (C-3), 70.5 (C-5), 69.9 (d, $^2J_{C,P}$ = 5.1 Hz, 1C, *C*$H_2$Ph), 69.8 (d, $^2J_{C,P}$ = 5.5 Hz, 1C, *C*$H_2$Ph), 67.0 (d, $^3J_{C,P}$ = 11.2 Hz, 1C, C-2), 66.6 (C-4), 62.1 (C-6), 57.9 (O$CH_3$), 20.8(1) (C(O)$CH_3$), 20.8 (C(O)$CH_3$), 20.6 (C(O)$CH_3$). $^{31}$P NMR (202 MHz, $CDCl_3$) δ −1.91. HRMS (ESI) *m/z* Calcd for $C_{27}H_{33}O_{12}PNa$ $[M + Na]^+$: 603.1602. Found: 603.1595.

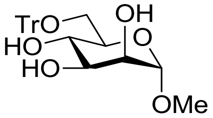


**Methyl 6-*O*-trityl-α-D-mannopyranoside (25)**

To a solution of methyl α-D-mannopyranoside **24** (3.9 g, 0.02 mol) in dry pyridine (100 mL) was added trityl chloride (6.7 g, 0.024 mol) and DMAP (0.47 g, 4 mmol). The solution was stirred at 40 °C overnight and then cooled. Evaporation of the solvent, followed by chromatography of the

residue (hexane–EtOAc 1:1 → 1:3) afforded **25** (7.4 g, 85%) as a colorless syrup $[\alpha]_D$ = +21.9 (*c* 0.6, $CHCl_3$); $^1$H NMR (500 MHz, $CDCl_3$) δ 7.71–7.08 (m, 15H, ArH), 4.72 (d, *J* = 1.3 Hz, 1H, H-1), 3.96–3.87 (m, 1H, H-2), 3.79 (dd, *J* = 8.9, 3.4 Hz, 1H, H-3), 3.73 (app t, *J* = 9.3 Hz, 1H, H-4), 3.69–3.62 (m, 1H, H-5), 3.46 (d, *J* = 4.8 Hz, 1H, H-6a), 3.42 (dd, *J* = 9.8, 5.3 Hz, 1H, H-6b), 3.38 (s, 3H, $OCH_3$). $^{13}$C NMR (126 MHz, $CDCl_3$) δ 143.6 (Ar), 128.6 (Ar), 128.0 (Ar), 127.2 (Ar), 100.6 (C-1), 87.4 ($OC(Ph)_3$), 71.6 (C-3), 70.4 70.3, 69.8 (C-2, C-4, C-5), 64.9 (C-6), 55.0 ($OCH_3$). HRMS (ESI) *m/z* Calcd for $C_{26}H_{28}O_6Na$ $[M + Na]^+$: 459.1778. Found: 459.1782.

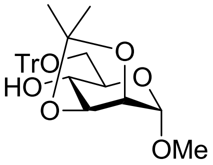


**Methyl 2,3-*O*-isopropylidene-6-*O*-trityl-α-D-mannopyranoside (26)**

Monosaccharide **25** (4.2 g, 9.6 mmol) was dissolved in 2,2-dimethoxypropane (20 mL). To this solution was added *p*-TsOH (183 mg, 0.96 mmol) and the reaction mixture was stirred overnight. Then, the solution was quenched by the addition of $NaHCO_3$ (satd aq soln). The solvent was evaporated and the residue was diluted with $CH_2Cl_2$, washed with distilled water and brine. The organic layer was dried over $Na_2SO_4$, concentrated and the residue was purified by chromatography (hexane–EtOAc 4:1) to give **26** (3.47 g, 76%) as a colorless oil. $[\alpha]_D$ = −1.3 (*c* 1.3, $CHCl_3$); $^1$H NMR (500 MHz, $CDCl_3$) δ 7.49–7.22 (m, 15H, ArH), 4.92 (s, 1H, H-1), 4.16–4.08 (m, 2H, H-2, H-3), 3.76–3.63 (m, 1H, H-4), 3.71–3.67 (m, 1H, H-5), 3.45–3.41 (m, 4H, H-6a, $OCH_3$), 3.38 (dd, *J* = 10.0, 5.2 Hz, 1H, H-6b), 2.45 (d, *J* = 3.8 Hz, 1H, O*H*), 1.50 (s, 3H, $CH_3$), 1.35 (s, 3H, $CH_3$). $^{13}$C NMR (125 MHz, $CDCl_3$) δ 143.7 (Ar), 128.7 (Ar), 127.9 (Ar), 127.2 (Ar), 109.5 ($CO_2(CH_3)_2$), 98.3 (C-1), 87.1 ($OC(Ph)_3$), 78.0 (C-3), 75.3 (C-2), 70.7 (C-4),

68.8 (C-5), 64.3 (C-6), 55.0 (O*C*$H_3$), 27.9 (*C*$H_3$), 26.1 (*C*$H_3$). HRMS (ESI) *m/z* Calcd for $C_{29}H_{32}O_6Na$ $[M + Na]^+$: 499.2091. Found: 499.2095.

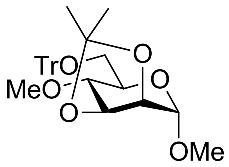


**Methyl 2,3-*O*-isopropylidene-4-*O*-methyl-6-*O*-trityl-α-D-mannopyranoside (27)**

Monosaccharide **26** (272 mg, 0.57 mmol) was dissolved in DMF (6 mL) and the solution was cooled to 0 °C. After the addition of NaH (60% NaH in mineral oil, 41 mg, 1.03 mmol), the mixture was stirred for 10 min before the addition of $CH_3I$ (53 μL, 0.86 mmol). The reaction mixture was stirred overnight and quenched by the addition of MeOH (1 mL). The solution was then diluted with $CH_2Cl_2$ and washed with distilled water and brine. The organic layer was dried over $Na_2SO_4$. Evaporation of the solvent and chromatography (hexane–EtOAc 10:1) of the residue afforded **27** (253 mg, 91%) as a colorless syrup. $^1H$ NMR (500 MHz, $CDCl_3$) δ 7.54–7.20 (m, 15H, ArH), 4.99 (s, 1H, H-1), 4.19–4.10 (m, 2H, H-3, H-2), 3.67-3.63 (m, 1H, H-5), 3.47 (s, 3H, OC*H*$_3$), 3.41 (dd, *J* = 9.9, 2.0 Hz, 1H, H-6a), 3.38 (dd, J = 10.2, 6.5 Hz, 1H, H-4), 3.32 (s, 1H, OC*H*$_3$), 3.20 (dd, *J* = 9.9, 5.4 Hz, 1H, H-6b), 1.58 (s, 1H, C*H*$_3$), 1.37 (s, 1H, C*H*$_3$). The $^1H$ spectral data was consistent with those previously reported [3]. HRMS (ESI) *m/z* Calcd for $C_{30}H_{34}O_6Na$ $[M + Na]^+$: 512.2248. Found: 513.2254.

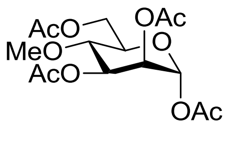


**1,2,3,6-Tetra-*O*-acetyl-4-*O*-methyl-α-D-mannopyranose (28)**

Monosaccharide **27** (247 mg, 0.5 mmol) was dissolved in $Ac_2O$–HOAc–$H_2SO_4$ (35:15:1 v/v/v, 3.06 mL). After being stirred overnight, the mixture was diluted with $CH_2Cl_2$ and washed with

distilled water, $NaHCO_3$ (satd aq soln) and brine. After drying over $Na_2SO_4$, the organic layer was concentrated and the crude residue purified by chromatography (hexane–EtOAc 2:1 → 1:1) to afford **28** (96 mg, 55%) as a pale yellow syrup. $[\alpha]_D$ = +42.5 (*c* 0.6, $CHCl_3$); $^1$H NMR (500 MHz, $CDCl_3$) δ 6.03 (d, *J* = 1.8 Hz, 1H, H-1), 5.24 (dd, *J* = 9.7, 3.5 Hz, 1H, H-3), 5.22 (dd, *J* = 3.5, 2.0 Hz, 1H, H-2), 4.36–4.28 (m, 2H, H-6a, H-6b), 3.91-3.87 (m, 1H, H-5), 3.56 (app t, *J* = 9.7 Hz, 1H, H-4), 3.47 (s, 3H, $OCH_3$), 2.16 (s, 3H, C(O)$CH_3$), 2.15 (s, 3H, C(O)$CH_3$), 2.12 (s, 3H, C(O)$CH_3$), 2.08 (s, 3H, C(O)$CH_3$). $^{13}$C NMR (125 MHz, $CDCl_3$) δ 171.7 (*C*=O), 169.8 (*C*=O), 169. 7 (*C*=O), 168.3 (*C*=O), 90. 7 (C-1, $^1J_{C,H}$ = 177.6 Hz), 74.3 (C-4), 71.8 (C-5), 71.3 (C-3), 68.7 (C-2), 62.9 (C-6), 60.7 ($OCH_3$), 20.9 (C(O)$CH_3$ × 2), 20.8 (C(O)$CH_3$), 20.8 (C(O)$CH_3$). HRMS (ESI) *m/z* Calcd for $C_{15}H_{22}O_{10}Na$ $[M + Na]^+$: 385.1105. Found: 385.1104.

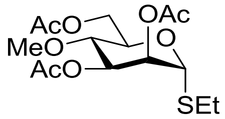


**Ethyl 2,3,6-tri-*O*-acetyl-4-*O*-methyl-1-thio-α-D-mannopyranoside (29)**

Tetraacetate **28** (259 mg, 0.72 mmol) and ethanethiol (80 µL, 1.08 mmol) were dissolved in $CH_2Cl_2$ (4 mL) and cooled to 0 °C before the addition of $BF_3{\cdot}OEt_2$ (180 µL, 1.44 mmol). The reaction mixture was stirred overnight and then was diluted with $CH_2Cl_2$, and washed with $NaHCO_3$ (satd aq soln), distilled water and brine. The organic layer was dried over $Na_2SO_4$, concentrated and the crude residue was purified by chromatography (hexane–EtOAc 2:1 → 1:1) to afford **29** (183 mg, 70%) as a pale yellow syrup. $[\alpha]_D$ = +75.0 (*c* 0.5, $CHCl_3$); $^1$H NMR (500 MHz, $CDCl_3$) δ 5.32 (dd, *J* = 3.4, 1.6 Hz, 1H, H-2), 5.23 (d, *J* = 1.5 Hz, 1H, H-1), 5.19 (dd, *J* = 9.6, 3.4 Hz, 1H, H-3), 4.41–4.30 (m, 2H, H-6a, H-6b), 4.25 (ddd, *J* = 9.9, 5.1, 2.3 Hz, 1H, H-5), 3.54 (app t, *J* = 9.7 Hz, 1H, H-4), 3.45 (s, 3H, $OCH_3$), 2.77–2.53 (m, 2H, $SCH_2CH_3$), 2.15 (s, 3H, C(O)$CH_3$), 2.12 (s, 3H, C(O)$CH_3$), 2.06 (s, 3H, C(O)$CH_3$), 1.29 (t, *J* = 7.4 Hz, 3H, $SCH_2CH_3$).

$^{13}$C NMR (125 MHz, $CDCl_3$) δ 170.7 (*C*=O), 169.9 (*C*=O), 169.6 (*C*=O), 82.1 (C-1), 74.9 (C-4), 71.9 (C-3), 71.6 (C-2), 70.0 (C-5), 63.2 (C-6), 60.4 (O*C*$H_3$), 25.4 (S*C*$H_2CH_3$), 21.0 (C(O)*C*$H_3$), 20.9 (C(O)*C*$H_3$), 20.8 (C(O)*C*$H_3$), 14.8 ($SCH_2$*C*$H_3$). HRMS (ESI) *m/z* Calcd for $C_{15}H_{24}O_8SNa$ $[M + Na]^+$: 387.1084. Found: 387.1084.

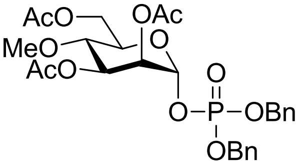


**Dibenzyl 2,3,6-tri-*O*-acetyl-4-*O*-methyl-α-D-mannopyranosyl phosphate (30)**

Thioglycoside **29** (178 mg, 0.49 mmol), dibenzyl phosphate (231 mg, 0.83 mmol), and powdered 4 Å molecular sieves were dissolved in dry $CH_2Cl_2$ and stirred for 30 min. Then, *N*–iodosuccinimide (165 mg, 0.74 mmol) and silver trifluoromethanesulfonate (38 mg, 0.15 mmol) were added at −40 °C. The reaction mixture was stirred for 3 h at −40 °C to −30 °C, and was then filtered through Celite and diluted with $CH_2Cl_2$. The solution was washed with $Na_2S_2O_3$ (satd aq soln), $NaHCO_3$ (satd aq soln), distilled water and brine. The organic layer was dried over $Na_2SO_4$, concentrated and the crude residue was purified by chromatography (hexane–EtOAc 1:1) to give **30** (226 mg, 80%) as a colorless oil. $[\alpha]_D$ = +37.4 (*c* 0.6, $CHCl_3$); $^1$H NMR (500 MHz, $CDCl_3$) δ 7.40–7.35 (m, 10H, ArH), 5.63 (dd, *J* = 6.6, 1.6 Hz, 1H, H-1), 5.29–5.26 (m, 2H, H-2, H-3), 5.14–5.06 (m, 4H, C*H*$_2$Ph), 4.26 (dd, *J* = 12.1, 4.8 Hz, 1H, H-6a), 4.21 (dd, *J* = 12.1, 2.3 Hz, 1H, H-6b), 3.98 (ddd, *J* = 9.6, 4.6, 2.3 Hz, 1H, H-5), 3.58–3.51 (m, 1H, H-4), 3.47 (s, 3H, OC*H*$_3$), 2.16 (s, 3H, C(O)C*H*$_3$), 2.09 (s, 3H, C(O)C*H*$_3$), 2.03 (s, 3H, C(O)C*H*$_3$). $^{13}$C NMR (126 MHz, $CDCl_3$) δ 170.6 (*C*=O), 169.6 (*C*=O), 169.5 (*C*=O), 135.4 (d, $^3J_{C,P}$ = 6.9 Hz, 1C, Ar), 135.3 (d, $^3J_{C,P}$ = 6.8 Hz, 1C, Ar), 128.7(3) (Ar), 128.7(1) (Ar), 128.6(8) (Ar), 128.2 (Ar), 128.0 (Ar), 95.3 (d, $^2J_{C,P}$ = 5.3 Hz, 1C, C-1, $^1J_{C,H}$ = 178.7 Hz), 74.0 (C-4), 71.4 (C-5), 70.7 (C-3),

69.9 (d, ${}^{2}J_{C,P}$ = 5.5 Hz, 1C, *C*H$_2$Ph), 69.7 (d, ${}^{2}J_{C,P}$ = 5.5 Hz, 1C, *C*H$_2$Ph), 69.2 (d, ${}^{3}J_{C,P}$ = 11.2 Hz, 1C, C-2), 62.7 (C-6), 60.6 (O*C*H$_3$), 20.9 (C(O)*C*H$_3$), 20.8 (C(O)*C*H$_3$), 20.7 (C(O)*C*H$_3$). HRMS (ESI) *m/z* Calcd for $C_{27}H_{33}O_{12}PNa$ $[M + Na]^+$: 603.1602. Found: 603.1603.

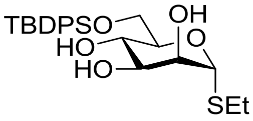


**Ethyl 6-*O*-*t*-butyldiphenylsilyl-1-thio-α-D-mannopyranoside (32)**

Thioglycoside **31** (5 g, 12.7 mmol) was dissolved in methanol (100 mL) and 1.0 M NaOMe (2 mL) was added dropwise. After stirring overnight, the reaction mixture was neutralized with Amberlite IR120 $H^+$ ion exchange resin and then concentrated. *tert*-Butylchlorodiphenylsilane (5.6 mL, 21.7 mmol) was added to a solution of the crude residue and imidazole (3.9 g, 57.3 mmol) in dry DMF (24 mL). The reaction mixture was stirred overnight and then quenched by the addition of MeOH (5 mL). The solvent was then evaporated and the resulting residue purified by chromatography (hexane–EtOAc 3:1 → 1:1) to provide **32** (4.6 g, 78%) as a colorless syrup. $[\alpha]_D$ = +88.0 (*c* 1.1, $CH_3OH$); ${}^{1}$H NMR (500 MHz, $CD_3OD$) δ 7.77–7.67 (m, 4H, ArH), 7.47–7.33 (m, 6H, ArH), 5.30 (d, *J* = 1.0 Hz, 1H, H-1), 4.10–4.04 (m, 1H, H-5), 4.02 (dd, *J* = 10.9, 2.0 Hz, 1H, H-6a), 3.90 (dd, *J* = 3.4, 1.4 Hz, 1H, H-2), 3.84 (dd, *J* = 10.9, 7.0 Hz, 1H, H-6b), 3.68 (dd, *J* = 9.4, 3.4 Hz, 1H, H-3), 3.59 (app t, *J* = 9.6 Hz, 1H, H-4), 2.78–2.50 (m, 2H, SC*H*$_2$CH$_3$), 1.28 (t, *J* = 7.4 Hz, 3H, SCH$_2$C*H*$_3$), 1.03 (s, 9H, Si(Ph)$_2$C(C*H*$_3$)$_3$ × 3). ${}^{13}$C NMR (125 MHz, $CD_3OD$) δ 136.8(4) (Ar), 136.8(0) (Ar), 134.9 (Ar), 134.8 (Ar), 130.7(9) (Ar), 130.7(5) (Ar), 128.7(3) (Ar), 128.7(0) (Ar), 85.4 (C-1), 75.4 (C-5), 73.5 (C-2), 73.4 (C-3), 69.2 (C-4), 65.3 (C-6), 27.3 (Si(Ph)$_2$C(*C*H$_3$)$_3$), 25.4 (S*C*H$_2$CH$_3$), 20.1 (Si(Ph)$_2$*C*(CH$_3$)$_3$), 15.2 (SCH$_2$*C*H$_3$). HRMS (ESI) *m/z* Calcd for $C_{24}H_{34}O_5SSiNa$ $[M + Na]^+$: 485.1788. Found: 485.1780.

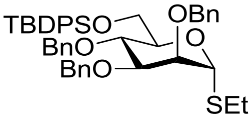


**Ethyl 2,3,4-tri-*O*-benzyl-6-*O*-(*tert*-butyldiphenylsilyl)-1-thio-α-D-mannopyranoside (33)**

Thioglycoside **32** (4.6 g, 9.9 mmol) was dissolved in dry THF (40 mL) and cooled to 0 °C. Then NaH (60% NaH in mineral oil, 2.3 g, 59.4 mmol) was added. The reaction mixture was stirred for 0.5 h before the addition of BnBr (5.3 mL, 44.6 mmol) and tetra-*n*-butylammonium iodide (0.37 g, 1 mmol). The reaction mixture was stirred overnight and then was quenched by the addition of MeOH. The solution was then diluted with $CH_2Cl_2$, washed with distilled water, $NaHCO_3$ (satd aq soln) and brine. The organic layer was dried over $Na_2SO_4$, and concentrated and the crude residue purified by chromatography (hexane–EtOAc 18:1) to provide **33** (6.1 g, 84%) as a pale yellow syrup. $[\alpha]_D$ = +33.8 (*c* 1.2, $CHCl_3$); $^1$H NMR (400 MHz, $CDCl_3$) δ 7.87–7.12 (m, 25H, ArH), 5.39 (d, $J$ = 1.3 Hz, 1H, H-1), 4.93–4.47 (m, 6H, C$H_2$Ph), 4.13–3.97 (m, 3H, H-4, H-5, H-6a), 3.90 (m, 2H, H-6b, H-3), 3.86–3.83 (m, 1H, H-2), 2.68–2.49 (m, 2H, SC$H_2$CH$_3$), 1.24 (t, $J$ = 7.4 Hz, 3H, SCH$_2$C$H_3$), 1.07 (s, 9H, Si(Ph)$_2$C(C$H_3$)$_3$). $^{13}$C NMR (125 MHz, $CDCl_3$) δ 138.6 (Ar), 138.4(0) (Ar), 138.3(8) (Ar), 136.0 (Ar), 135.7 (Ar), 133.9 (Ar), 133.4 (Ar), 129.5(1) (Ar), 129.4(9) (Ar), 128.3(9) (Ar), 128.3(5) (Ar), 128.3(1) (Ar), 128.0 (Ar), 127.9 (Ar), 127.8 (Ar), 127.7(4) (Ar), 127.6(6) (Ar), 127.6(5) (Ar), 127.6(3) (Ar), 127.5(7) (Ar), 127.5(2) (Ar), 81.4 (C-1), 80.6 (C-3), 77.0 (C-2), 75.2 (*C*H$_2$Ph), 75.0 (C-4), 73.4 (C-5), 72.2(4) (*C*H$_2$Ph), 72.2(0) (Ar*C*H$_2$), 63.3 (C-6), 26.8 (Si(Ph)$_2$C(*C*H$_3$)$_3$), 25.0 (S*C*H$_2$), 19.3 (Si(Ph)$_2$*C*(CH$_3$)$_3$), 14.8 (SCH$_2$*C*H$_3$). HRMS (ESI) *m/z* Calcd for $C_{45}H_{52}O_5SSiNa$ $[M + Na]^+$: 755.3197. Found: 755.3196.

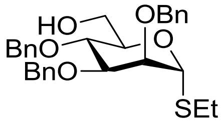

**Ethyl 2,3,4-tri-*O*-benzyl-1-thio-α-D-mannopyranoside (34)**

A 1 M solution of tetra-*n*-butylammonium fluoride in THF (13.4 mL, 13.4 mmol) was added dropwise to a solution of **33** (6.07 g, 8.3 mmol) in THF (29 mL), and the mixture was stirred overnight. Evaporation of the solvent and chromatography (hexane–EtOAc 7:2) of the crude residue provided the alcohol **34** (3.4 g, 83%) as colorless oil. $[\alpha]_D$ = +81.0 (*c* 0.8, $CHCl_3$); $^1$H NMR (400 MHz, $CDCl_3$) δ 7.42–7.27 (m, 15H, ArH), 5.31 (d, *J* = 1.2 Hz, 1H, H-1), 4.95–4.57 (m, 6H, $ArCH_2$), 4.04–3.98 (m, 2H, H-4, H-5), 3.89–3.79 (m, 4H, H-2, H-3, H-6a, H-6b), 2.67–2.48 (m, 2H, $SCH_2CH_3$), 1.93 (app t, *J* = 6.5 Hz, 1H, OH), 1.24 (t, *J* = 7.4 Hz, 3H, $SCH_2CH_3$). $^{13}$C NMR (101 MHz, $CDCl_3$) δ 138.4 (Ar), 138.2 (Ar), 138.1 (Ar), 128.4 (Ar), 128.1 (Ar), 127.9 (Ar), 127.8 (Ar), 127.7(2) (Ar), 127.6(8) (Ar), 82.3 (C-1), 80.4 (C-3), 76.6 (C-2), 75.2 ($CH_2Ph$), 75.0 (C-4), 72.4(1) (C-5), 72.3(9) ($CH_2Ph$), 72.2 ($CH_2Ph$), 62.4 (C-6), 25.4 ($SCH_2$), 14.9 ($SCH_2CH_3$). The $^1$H and $^{13}$C NMR spectral data were consistent with those previously reported [4,5]. HRMS (ESI) *m/z* Calcd for $C_{29}H_{34}O_5SNa$ $[M + Na]^+$: 517.2019. Found: 517.2012.

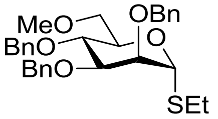

**Ethyl 2,3,4-tri-*O*-benzyl-6-*O*-methyl-1-thio-α-D-mannopyranoside (35)**

Monosaccharide **34** (148.4 mg, 0.3 mmol) was dissolved in DMF (3 mL) and the solution was cooled to 0 °C. After the addition of NaH (60% NaH in mineral oil, 20.4 mg, 5.1 mmol), the mixture was stirred for 10 min before the addition of $CH_3I$ (26 µL, 0.42 mmol). The reaction mixture was stirred overnight and quenched by the addition of MeOH (1 mL). The solution was then diluted with $CH_2Cl_2$, washed with distilled water and brine. The organic layer was dried

over $Na_2SO_4$. Evaporation of the solvent and chromatography (hexane–EtOAc 8:1) of the residue afforded **35** (133 mg, 87%) as a colorless syrup $[\alpha]_D$ = +82.1 (*c* 0.6, $CHCl_3$); $^1H$ NMR (400 MHz, $CDCl_3$) δ 7.42–7.26 (m, 15H, ArH), 5.39 (s, 1H, H-1), 4.94–4.50 (m, 6H, $CH_2Ph$), 4.09 (ddd, *J* = 9.8, 4.7, 1.8 Hz, 1H, H-5), 4.01 (app t, *J* = 9.3 Hz, 1H, H-4), 3.86–3.81 (m, 2H, H-2, H-3), 3.70 (dd, *J* = 10.6, 4.8 Hz, 1H, H-6), 3.60 (dd, *J* = 10.6, 2.0 Hz, 1H, H-6), 3.39 (s, 3H, $OCH_3$), 2.69–2.51 (m, 2H, $SCH_2CH_3$), 1.25 (t, *J* = 7.4 Hz, 1H, $SCH_2CH_3$). $^{13}C$ NMR (101 MHz, $CDCl_3$) δ 138.6 (Ar), 138.3 (Ar), 138.1 (Ar), 128.3 (Ar), 128.0 (Ar), 127.9 (Ar), 127.7(2) (Ar), 127.6(5) (Ar), 127.6 (Ar), 82.0 (C-1), 80.3 (C-3), 76.3 (C-2), 75.1(4) ($CH_2Ph$), 75.0(5) (C-4), 72.0(1) ($CH_2Ph$), 71.9(9) ($CH_2Ph$), 71.7 (C-5), 71.6 (C-6), 59.2 ($OCH_3$), 25.3 ($SCH_2CH_3$), 14.9 ($SCH_2CH_3$). HRMS (ESI) *m/z* Calcd for $C_{30}H_{36}O_5SNa$ $[M + Na]^+$: 531.2176. Found: 531.2169.

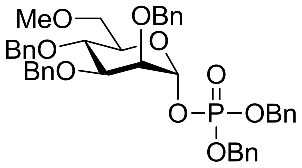


**Dibenzyl 2,3,4-tri-*O*-benzyl-6-*O*-methyl-α-D-mannopyranosyl phosphate (36)**

Thioglycoside **35** (50 mg, 0.1 mmol), dibenzyl phosphate (47.3 mg, 0.17 mmol), and powdered 4 Å molecular sieves were dissolved in dry $CH_2Cl_2$ (3 mL) and stirred for 40 min. Then, *N*-iodosuccinimide (34 mg, 0.15 mmol) and silver trifluoromethanesulfonate (8 mg, 0.03 mmol) were added at −30 °C. The reaction mixture was stirred for 40 min from −30 °C to −20 °C, and then was filtered through Celite and diluted with $CH_2Cl_2$. The solution was washed with $Na_2S_2O_3$ (satd aq soln), $NaHCO_3$ (satd aq soln), distilled water and brine. The organic layer was dried over $Na_2SO_4$, and concentrated. The crude residue was purified by chromatography (hexane–EtOAc 5:2) to afford **36** (50.4 mg, 70%) as a colorless oil. $[\alpha]_D$ = +24.8 (*c* 1.3, $CHCl_3$); $^1H$ NMR (500 MHz, $CDCl_3$) δ 7.48–7.27 (m, 25H, ArH), 5.86 (dd, *J* = 6.1, 1.9 Hz, 1H, H-1), 5.21–4.47

(m, 10H, C$H_2$Ph × 5), 4.11 (app t, $J$ = 9.7 Hz, 1H, H-4), 3.95 (ddd, $J$ = 10.0, 4.3, 1.5 Hz, 1H, H-5), 3.90 (dd, $J$ = 9.5, 3.0 Hz, 1H, H-3), 3.80 (app t, $J$ = 2.5 Hz, 1H, H-2), 3.68 (dd, $J$ = 10.9, 4.5 Hz, 1H, H-6a), 3.55 (dd, $J$ = 10.8, 1.7 Hz, 1H, H-6b), 3.39 (s, 3H, OC$H_3$). $^{13}$C NMR (125 MHz, $CDCl_3$) δ 138.5 (Ar), 138.3 (Ar), 137.6, (Ar), 137.8 (d, $^3J_{C,P}$ = 6.9 Hz, 1C, Ar), 135.7 (d, $^3J_{C,P}$ = 7.0 Hz, 1C, Ar), 128.6(2) (Ar), 128.5(9) (Ar), 128.4(1) (Ar), 128.3(9) (Ar), 128.0(1) (Ar), 127.9(9) (Ar), 127.9 (Ar), 127.8 (Ar), 127.7(3) (Ar), 127.7(1) (Ar), 127.6(5) (Ar), 127.6(2) (Ar), 96.5 (d, $^2J_{C,P}$ = 6.3 Hz, 1C, C-1, $^1J_{C,H}$ = 176.4 Hz), 78.9 (C-3), 75.2 (C$H_2$Ph), 74.4 (d, $^3J_{C,P}$ = 9.0 Hz, 1C, C-2), 74.1 (C-4), 73.7 (C-5), 72.8 ($CH_2$Ph), 72.2 ($CH_2$Ph), 71.1 (C-6), 69.5 ($CH_2$Ph), 69.4 ($CH_2$Ph), 59.3 (O$CH_3$). $^{31}$P NMR (202 MHz, $CDCl_3$) δ −1.65. HRMS (ESI) *m/z* Calcd for $C_{42}H_{45}O_9PNa$ [M + Na]$^+$: 747.2693. Found: 747.2685.

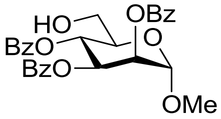


**Methyl 2,3,4-tri-*O*-benzoyl-α-D-mannopyranoside (37)**

Monosaccharide methyl 2,3,4-tri-*O*-benzoyl-6-*O*-trityl-α-D-mannopyranoside **[6]** (749 mg, 1 mmol) was dissolved in 1:1 $CH_2Cl_2$–MeOH (10 mL). To this solution was added 10% HCl in MeOH (2 mL, HCl: MeOH v/v = 1:9). The solution was stirred overnight and then the solvent was evaporated. The residue was purified by chromatography (hexane–EtOAc 2:1 → 1:2) to provide **37** (410 mg, 81%) as a colorless syrup $[\alpha]_D$ = −156.7 (*c* 0.5, $CHCl_3$); $^1$H NMR (500 MHz, $CDCl_3$) δ 8.17–7.22 (m, 15H, ArH), 5.98 (dd, $J$ = 10.1, 3.4 Hz, 1H, H-3), 5.86 (app t, $J$ = 10.1 Hz, 1H, H-4), 5.69 (dd, $J$ = 3.3, 1.8 Hz, 1H, H-2), 5.02 (d, $J$ = 1.5 Hz, 1H, H-1), 4.09 (ddd, $J$ = 10.0, 3.9, 2.2 Hz, 1H, H-5), 3.86 (dd, $J$ = 12.7, 2.2 Hz, 1H, H-6a), 3.80 (dd, $J$ = 12.7, 4.0 Hz, 1H, H-6b), 3.53 (s, 3H, OC$H_3$), 2.62 (dd, $J$ = 8.4, 5.8 Hz, 1H, O$H$). $^{13}$C NMR (126 MHz, $CDCl_3$) δ 166.5 ($C$=O), 165.6 ($C$=O), 165.5 ($C$=O), 133.7 (Ar), 133.6 (Ar), 133.2 (Ar), 129.9(4) (Ar),

129.9(2) (Ar), 129.7(2) (Ar), 129.3(3) (Ar), 129.2 (Ar), 128.8 (Ar), 128.7 (Ar), 128.5 (Ar), 128.3 (Ar), 98.8 (C-1), 70.9 (C-5), 70.6 (C-2), 69.6 (C-3), 67.3 (C-4), 61.4 (C-6), 55.5 (O*C*$H_3$). HRMS (ESI) *m/z* Calcd for $C_{28}H_{26}O_9Na$ $[M + Na]^+$: 529.1469. Found: 529.1461.

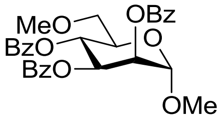


**Methyl 2,3,4-tri-*O*-benzoyl-6-*O*-methyl-α-D-mannopyranoside (38)**

Monosaccharide **37** (112.8 mg, 0.22 mmol) was dissolved in $CH_3I$ (2.5 mL). To this solution was added $Ag_2O$ (155 mg, 0.67 mmol) and $CaSO_4$ (121 mg, 0.89 mmol). The reaction mixture was stirred for 3 days, and then filtered through Celite. The filtrate was concentrated and the crude residue was purified by chromatography (hexane–EtOAc 4:1) to afford **38** (60 mg, 52%) as a colorless syrup, as well as the side product methyl 2,3,6-tri-*O*-benzoyl-4-*O*-methyl-α-D-mannopyranoside (35 mg, 31%) as a colorless syrup. $^1$H NMR (500 MHz, $CDCl_3$) δ 8.15–7.20 (m, 15H, ArH), 5.91–5.85 (m, 2H, H-4, H-3), 5.66 (dd, *J* = 2.8, 1.8 Hz, 1H, H-2), 5.00 (d, *J* = 1.7 Hz, 1H, H-1), 4.24-4.23 (m, 1H, H-5), 3.64 (m, 2H, H-6a, H-6b), 3.54 (s, 3H, OC$H_3$), 3.40 (s, 3H, OC$H_3$). $^{13}$C NMR (125 MHz, $CDCl_3$) δ 165.5(7) (*C*=O), 165.5(6) (*C*=O), 165.4 (*C*=O), 133.4 (Ar), 133.3 (Ar), 133.1 (Ar), 129.9 (Ar), 129.8 (Ar), 129.7 (Ar), 129.4 (Ar), 129.3 (Ar), 129.2 (Ar), 128.5 (Ar), 128.4 (Ar), 128.2 (Ar), 98.6 (C-1), 71.6 (C-6), 70.5 (C-2), 70.0 (C-5), 69.9, 67.4 (C-4, C-3), 59.6 (O*C*$H_3$), 55.5 (O*C*$H_3$). The $^1$H and $^{13}$C NMR spectral data were consistent with those previously reported [7]. HRMS (ESI) *m/z* Calcd for $C_{29}H_{28}O_9Na$ $[M + Na]^+$: 543.1626. Found: 543.1618.

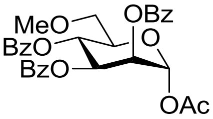


**1-*O*-Acetyl-2,3,4-tri-*O*-benzoyl-6-*O*-methyl-α-D-mannopyranose (39)**

Monosaccharide **38** (286 mg, 0.55 mmol) was dissolved in $Ac_2O$–HOAc–$H_2SO_4$ (70:30:1 v/v/v, 5.05 mL). After being stirred overnight, the mixture was diluted with $CH_2Cl_2$ and washed with distilled water, $NaHCO_3$ (satd aq soln) and brine. The organic layer was dried over $Na_2SO_4$ and concentrated, and the crude residue was purified by chromatography (hexane–EtOAc 2:1) to afford **39** (289 mg, 96%) as a pale yellow syrup $[\alpha]_D = -130.2$ (*c* 0.9, $CHCl_3$); $^1$H NMR (400 MHz, $CDCl_3$) δ 8.24–7.15 (m, 15H, ArH), 6.39 (d, *J* = 2.0 Hz, 1H, H-1), 5.98 (app t, *J* = 10.0 Hz, 1H, H-4), 5.89 (dd, *J* = 10.2, 3.3 Hz, 1H, H-3), 5.71 (dd, *J* = 3.3, 2.0 Hz, 1H, H-2), 4.31 (app dt, *J* = 9.9, 3.9 Hz, 1H, H-5), 3.65 (d, *J* = 4.0 Hz, 2H, H-6a, H-6b), 3.38 (s, 3H, $OCH_3$), 2.26 (s, 3H, $C(O)CH_3$). $^{13}$C NMR (126 MHz, $CDCl_3$) δ 168.2 (*C*=O), 165.6 (*C*=O), 165.4 (*C*=O), 165.3 (*C*=O), 133.6 (Ar), 133.4 (Ar), 133.3 (Ar), 130.0 (Ar), 129.7(8) (Ar), 129.7(6) (Ar), 129.0(8) (Ar), 129.0(6) (Ar), 128.9 (Ar), 128.6 (Ar), 128.5 (Ar), 128.4 (Ar), 90.9 (C-1, $^1J_{C,H}$ = 178.5 Hz), 72.4 (C-5), 71.4 (C-6), 69.8 (C-3), 69.3 (C-2), 66.9 (C-4), 59.7 ($OCH_3$), 21.0 ($C(O)CH_3$). HRMS (ESI) *m/z* Calcd for $C_{30}H_{28}O_{10}Na$ $[M + Na]^+$: 571.1575. Found: 571.1579.

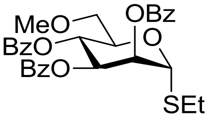


**Ethyl 2,3,4-tri-*O*-benzoyl-6-*O*-methyl-1-thio-α-D-mannopyranoside (40)**

Monosaccharide **39** (280 mg, 0.51 mmol) and ethanethiol (57 µL, 0.77 mmol) were dissolved in $CH_2Cl_2$ (4 mL) and cooled to 0 °C before the addition of $BF_3{\cdot}OEt_2$ (128 µL, 1.02 mmol). The reaction mixture was stirred overnight and then diluted with $CH_2Cl_2$, and washed with $NaHCO_3$ (satd aq soln), distilled water, and brine. The organic layer was dried over $Na_2SO_4$, concentrated

and the crude residue purified by chromatography (hexane–EtOAc 5:1 → 4:1) to afford **40** (211 mg, 75%) as a pale yellow syrup $[\alpha]_D$ = −49.3 (*c* 1.4, $CHCl_3$); $^1H$ NMR (500 MHz, $CDCl_3$) δ 8.12–8.10 (m, 2H, ArH), 8.00–7.98 (m, 2H, ArH), 7.84–7.82 (m, 2H, ArH), 7.61–7.57 (m, 1H, ArH), 7.53–7.45 (m, 3H, ArH), 7.43–7.36 (m, 3H, ArH), 7.27–7.23 (m, 2H, ArH), 5.96 (app. t, *J* = 10.0 Hz, 1H, H-4), 5.81 (dd, *J* = 9.9, 3.3 Hz, 1H, H-3), 5.78 (dd, *J* = 3.3, 1.6 Hz, 1H, H-2), 5.58 (d, *J* = 1.4 Hz, 1H, H-1), 4.62 (ddd, *J* = 9.9, 4.8, 3.0 Hz, 1H, H-5), 3.69–3.61 (m, 2H, H-6a, H-6b), 3.39 (s, 3H, $OCH_3$), 2.83–2.68 (m, 2H, $SCH_2CH_3$), 1.37 (t, *J* = 7.4 Hz, 3H, $SCH_2CH_3$). $^{13}C$ NMR (125 MHz, $CDCl_3$) δ 165.5(3) (*C*=O), 165.5(1) (*C*=O), 165.4 (*C*=O), 133.5 (Ar), 133.4 (Ar), 133.2 (Ar), 129.9 (Ar), 129.8 (Ar), 129.7 (Ar), 128.6 (Ar), 128.5 (Ar), 128.3 (Ar), 82.2 (C-1), 72.3 (C-2), 71.4 (C-6), 70.6 (C-3), 70.4 (C-5), 67.6 (C-4), 59.6 ($OCH_3$), 25.5 ($SCH_2CH_3$), 14.9 ($SCH_2CH_3$). HRMS (ESI) *m/z* Calcd for $C_{30}H_{30}O_8SNa$ $[M + Na]^+$: 573.1554. Found: 573.1544.

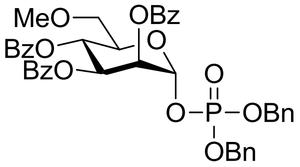


**Dibenzyl 2,3,4-tri-*O*-benzoyl-6-*O*-methyl-α-D-mannopyranosyl phosphate (41)**

Thioglycoside **40** (103 mg, 0.18 mmol), dibenzyl phosphate (85 mg, 0.31 mmol), and powdered 4 Å molecular sieves were dissolved in dry $CH_2Cl_2$ and stirred for 30 min. Then, *N*-iodosuccinimide (61 mg, 0.27 mmol) and silver trifluoromethanesulfonate (14 mg, 0.054 mmol) were added at –30 °C. The reaction mixture was stirred for 2 h, and then was filtered through Celite. The filtrate was diluted with $CH_2Cl_2$, and washed with $Na_2S_2O_3$ (satd aq soln), $NaHCO_3$ (satd aq soln), distilled water and brine. The organic layer was dried over $Na_2SO_4$, concentrated and the crude residue was purified by chromatography (hexane–EtOAc 3:2) to afford **41** (123 mg, 89%) as a colorless oil $[\alpha]_D$ = −87.4 (*c* 2.0, $CHCl_3$); $^1H$ NMR (400 MHz, $CDCl_3$) δ 8.23–7.18

(m, 25H, ArH), 5.97 (app t, $J$ = 10.2 Hz, 1H, H-4), 5.95 (dd, $J$ = 6.2, 2.3 Hz, 1H, H-1), 5.86 (dd, $J$ = 10.1, 3.3 Hz, 1H, H-3), 5.69 (dd, $J$ = 3.2, 2.1 Hz, 1H, H-2), 5.24–5.17 (m, 4H, C$H_2$Ph × 2), 4.37 (app dt, $J$ = 10.0, 3.6 Hz, 1H, H-5), 3.57–3.53 (m, 2H, H-6a, H-6b), 3.34 (s, 3H, OC$H_3$). $^{13}$C NMR (126 MHz, $CDCl_3$) δ 165.3(5) (*C*=O), 165.3(2) (*C*=O), 165.1 (*C*=O), 135.6 (d, $^3J_{C,P}$ = 7.1 Hz, 1C, Ar), 135.4 (d, $^3J_{C,P}$ = 6.8 Hz, 1C, Ar), 133.6 (Ar), 133.4 (Ar), 133.2 (Ar), 130.0 (Ar), 129.8(1) (Ar), 129.7(6) (Ar), 129.1 (Ar), 129.0 (Ar), 128.7(4) (Ar), 128.7(0) (Ar), 128.6(6) (Ar), 128.6 (Ar), 128.5 (Ar), 128.3(2) (Ar), 128.2(7) (Ar), 128.1 (Ar), 127.9 (Ar), 95.4 (d, $^2J_{C,P}$ = 5.5 Hz, 1C, C-1, $^1J_{C,H}$ = 179.9 Hz), 71.9 (C-5), 70.8 (C-6), 70.0 (d, $^2J_{C,P}$ = 5.3 Hz, 1C, *C*H$_2$Ph), 69.9 (d, $^2J_{C,P}$ = 5.4 Hz, 1C, *C*H$_2$Ph), 69.8 (d, $^3J_{C,P}$ = 11.3 Hz, 1C, C-2), 69.3 (C-3), 66.6 (C-4), 59.5 (O*C*H$_3$). $^{31}$P NMR (162 MHz, $CDCl_3$) δ −2.97. HRMS (ESI) *m/z* Calcd for $C_{42}H_{39}O_{12}PNa$ [M + Na]$^+$: 789.2071. Found: 789.2063.

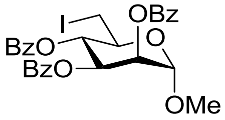


**Methyl 2,3,4-tri-*O*-benzoyl-6-deoxy-6-iodo-α-D-mannopyranoside (42)**

To a solution of **37** (3.25 g, 6.4 mmol) in dry $CH_2Cl_2$ (34 mL) was added $PPh_3$ (3.37 g, 12.8 mmol), imidazole (874 mg, 12.8 mmol) and $I_2$ (3.26 g, 12.8 mmol) at 0 °C. The solution was stirred overnight, diluted with $CH_2Cl_2$, and then washed with $Na_2S_2O_3$ (satd aq soln) and brine. The organic layer was dried over $Na_2SO_4$ and concentrated, and the residue was purified by chromatography (hexane–EtOAc 4:1) to provide **42** (2.56 g, 65%) as a pale yellow syrup. $^1$H NMR (500 MHz, $CDCl_3$) δ 8.20–7.20 (m, 15H, ArH), 5.89 (dd, $J$ = 10.0, 3.4 Hz, 1H, H-3), 5.77 (app t, $J$ = 9.8 Hz, 1H, H-4), 5.68 (dd, $J$ = 3.4, 1.8 Hz, 1H, H-2), 5.02 (d, $J$ = 1.7 Hz, 1H, H-1), 4.11–4.05 (m, 1H, H-5), 3.60 (s, 3H, OC$H_3$), 3.49 (dd, $J$ = 10.9, 2.6 Hz, 1H, H-6a), 3.38 (dd, $J$ = 10.9, 8.1 Hz, 1H, H-6b). $^{13}$C NMR (125 MHz, $CDCl_3$) δ 165.6 (*C*=O), 165.5 (*C*=O), 165.4

(*C*=O), 133.6(1) (Ar), 133.5(5) (Ar), 133.2 (Ar), 132.3 (Ar), 132.2 (Ar), 131.6 (Ar), 130.0 (Ar), 129.9 (Ar), 129.7 (Ar), 129.3 (Ar), 129.1 (Ar), 128.8 (Ar), 128.6 (Ar), 128.5 (Ar), 128.3 (Ar), 98.8 (C-1), 70.7, 70.5, 70.2, 69.6 (C-2, C-3, C-4, C-5), 55.8 (O*C*$H_3$), 4.7 (C-6). The $^1$H and $^{13}$C NMR spectral data were consistent with those previously reported [8]. HRMS (ESI) *m/z* Calcd for $C_{28}H_{25}IO_8Na$ [M + Na]$^+$: 639.0486. Found: 639.0492.

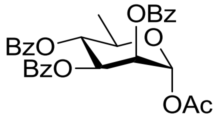


**1-*O*-Acetyl 2,3,4-tri-*O*-benzoyl-6-deoxy-α-D-mannopyranose (43)**

Monosaccharide **42** (867 mg, 1.4 mmol) was dissolved in $Ac_2O$–HOAc–$H_2SO_4$ (35:15:1 v/v/v, 9.2 mL). After being stirred overnight, the mixture was diluted with $CH_2Cl_2$ and washed with distilled water, $NaHCO_3$ (satd aq soln) and brine. The organic layer was dried over $Na_2SO_4$, concentrated and then dissolved in EtOAc (10 mL). To this solution was added 10% Pd–C (100 mg) and $Et_3N$ (1.5 mL). The reaction mixture was stirred for 2 days under a hydrogen atmosphere. The catalyst was then removed by filtration through Celite and the filtrate was concentrated. The residue was purified by chromatography (hexane–EtOAc 4:1 → 3:1) to afford **43** (525 mg, 72%) as a pale yellow syrup. $[\alpha]_D$ = −133.5 (*c* 0.6, $CHCl_3$); $^1$H NMR (500 MHz, $CDCl_3$) δ 8.14–7.24 (m, 15H, ArH), 6.32 (d, *J* = 1.6 Hz, 1H, H-1), 5.86 (dd, *J* = 10.2, 3.4 Hz, 1H, H-3), 5.76–5.70 (m, 2H, H-2, H-4), 4.28 (dq, *J* = 10.0, 6.0 Hz, 1H, H-5), 2.26 (s, 3H, C(O)C$H_3$), 1.40 (d, *J* = 6.2 Hz, 3H, H-6). $^{13}$C NMR (125 MHz, $CDCl_3$) δ 168.4 (*C*=O), 165.6 (*C*=O), 165.3 (*C*=O) 133.7 (Ar), 133.6 (Ar), 133.4 (Ar), 133.3 (Ar), 130.0 (Ar), 129.7(4) (Ar), 129.7(1) (Ar), 129.1(4) (Ar), 129.0(9) (Ar), 128.9(6) (Ar), 128.6 (Ar), 128.5 (Ar), 128.3 (Ar), 90.8 (C-1, $^1J_{C,H}$ = 177.2 Hz), 71.2 (C-4), 69.7 (C-2), 69.6 (C-3), 69.1 (C-5), 21.0 (C(O)*C*$H_3$), 17.7 (C-6). HRMS (ESI) *m/z* Calcd for $C_{29}H_{26}O_9Na$ [M + Na]$^+$: 541.1469. Found: 541.1465.

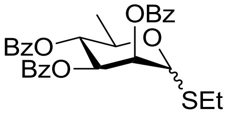


**Ethyl 2,3,4-tri-*O*-benzoyl-6-deoxy-1-thio-α/β-D-mannopyranoside (44)**

Monosaccharide **43** (361 mg, 0.70 mmol) and ethanethiol (78 µL, 1.05 mmol) were dissolved in $CH_2Cl_2$ (6 mL) and cooled to 0 °C before the addition of $BF_3{\cdot}OEt_2$ (176 µL, 1.40 mmol). The reaction mixture was stirred overnight and then was diluted with $CH_2Cl_2$, and washed with $NaHCO_3$ (satd aq soln), distilled water and brine. The organic layer was dried over $Na_2SO_4$ and concentrated, and the crude residue was purified by chromatography (hexane–EtOAc 6:1 → 5:1) to afford **44** (324 mg, 89%, α/β 4:1) as a pale yellow syrup. Data for α isomer: $^1$H NMR (500 MHz, $CDCl_3$) δ 8.26–7.17 (m, 15H, ArH), 5.85–5.78 (m, 2H, H-2, H-3), 5.78–5.71 (m, 1H, H-4), 5.52 (s, 1H, H-1), 4.59 (app dq, *J* = 9.5, 6.2 Hz, 1H, H-5), 2.87–2.59 (m, 2H, $SCH_2CH_3$), 1.41 (d, *J* = 6.3 Hz, 3H, H-6), 1.38 (t, *J* = 7.4 Hz, 3H, $SCH_2CH_3$). $^{13}$C NMR (126 MHz, $CDCl_3$) δ 165.8 (C=O), 165.6 (C=O), 165.4 (C=O), 133.5 (Ar), 133.4 (Ar), 133.1 (Ar), 129.9 (Ar), 129.8 (Ar), 129.7 (Ar), 129.5 (Ar), 129.3 (Ar), 129.1 (Ar), 128.6 (Ar), 128.5 (Ar), 128.3 (Ar), 82.2 (C-1), 72.7, 72.1, 70.5 (C-2, C-3, C-4), 67.4 (C-5), 25.7 ($SCH_2CH_3$), 17.6 (C-6), 15.0 ($SCH_2CH_3$). $^1$H and $^{13}$C NMR spectral data were consistent with those previously reported [9]. HRMS (ESI) *m/z* Calcd for $C_{29}H_{28}O_7SNa$ $[M + Na]^+$: 543.1448. Found: 543.1448.

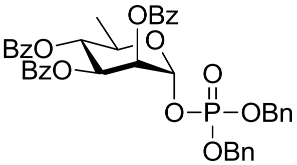


**Dibenzyl 2,3,4-tri-*O*-benzoyl-6-deoxy-α-D-mannopyranosyl phosphate (45)**

Thioglycoside **44** (64 mg, 0.12 mmol), dibenzyl phosphate (58 mg, 0.21 mmol), and powdered 4 Å molecular sieves were dissolved in dry $CH_2Cl_2$ (2 mL) and stirred at room temperature for 30

min. *N*–iodosuccinimide (42 mg, 0.19 mmol) and silver trifluoromethanesulfonate (10 mg, 0.039 mmol) were then added at −30 °C. The reaction mixture was stirred for 2 h at −30 °C, and then filtered through Celite and diluted with $CH_2Cl_2$. The solution was washed with $Na_2S_2O_3$ (satd aq soln), $NaHCO_3$ (satd aq soln), distilled water and brine. The organic layer was dried over $Na_2SO_4$, concentrated and the crude residue was purified by chromatography (hexane–EtOAc 2:1) to afford **45** (59 mg, 67%) as a colorless oil. $^1$H NMR (500 MHz, $CDCl_3$) δ 8.08 (m, 2H, ArH), 7.97 (m, 2H, ArH), 7.83 (m, 2H, ArH), 7.66–7.25 (m, 19H, ArH), 5.85 (dd, *J* = 6.3, 1.9 Hz, 1H, H-1), 5.80 (dd, *J* = 10.2, 3.4 Hz, 1H, H-3), 5.69–5.64 (m, 2H, H-2, H-4), 5.23–5.14 (m, 4H, C*H*$_2$Ph), 4.29 (dq, *J* = 9.9, 6.2 Hz, 1H, H-5), 1.28 (d, *J* = 6.2 Hz, 3H, H-6). $^{31}$P NMR (202 MHz, $CD_3Cl_3$) δ −1.76. HRMS (ESI) *m/z* Calcd for $C_{41}H_{37}O_{11}PNa$ $[M + Na]^+$: 759.1966. Found: 759.1960.

## Enzymatic synthesis of GDP-Man*p* analogues

GDP-α-D-mannose pyrophosphorylase from *Salmonella enterica* [10] was cloned, expressed in *E.coli* BL21(DE3)plySs, and purified. The enzymatic synthesis of GDP-Man*p* and GDP-Man*p* analogues were carried out in MOPS buffer (50 mM, pH 7.6) containing $MgCl_2$ (5 mM), DTT (1 mM), GTP (5 mM), Man*p*-1P or its analogues (5 mM), GDP-ManPP (200 μg), and inorganic pyrophosphatase (1.25 U) in a total reaction volume of 100 μL. The mixture was incubated at room temperature overnight with shaking and the reactions were stopped by removing the extra enzymes though centrifugal filtration when HPLC analysis of the spin-filtered (Amicon YM10) solution indicated that the reaction was complete.

## Characterization of GDP-Man*p* analogues

HPLC analysis was carried out by using a Waters 600E HPLC equipped with a photodiode array (PDA) detector, with monitoring at 262 nm; the system was controlled by Empower chromatography software. Monitoring the conversion of Man*p*-1P analogues into corresponding GDP-Man*p* analogues was done by analytical HPLC on a Phenomenex C18 column (4.6 × 250 mm). A gradient elution using two buffers, buffer A (200 mM $Et_3N$–HOAc, pH 6.6) and buffer B (200 mM $Et_3N$–HOAc pH 6.6 containing 5% $CH_3CN$), was used. The gradient conditions employed used 96% buffer A and 4% buffer B for 10 min (isocratic), followed by a gradient of 4→100% buffer B over 15 min, 100% buffer B for 10 min. The column was re-equilibrated with a gradient of 100→4% buffer B for 15 min, followed by 96% buffer A and 4% buffer B for 10 min. At the end of the reaction, the enzymes were removed by passing the incubation mixture through an empty 10 mL BioRad cartridge, equipped with a filter. The filtrate was then collected by passage through Amicon Ultra-15 centrifugal filter with a molecular-weight cut off of 10,000 Daltons; this filtrate was centrifuged to remove soluble proteins. Following centrifugation, the filtrate was collected and was applied to a Sephadex G-15 gel filtration column to remove salts.

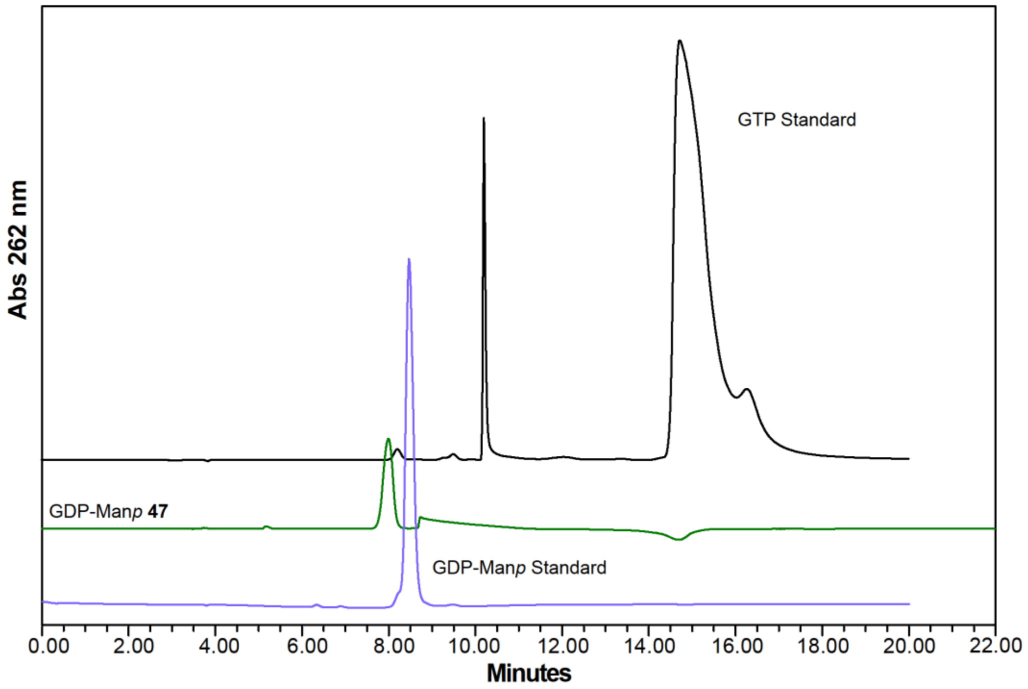


**Figure S1**. GDP-Man*p* HPLC traces

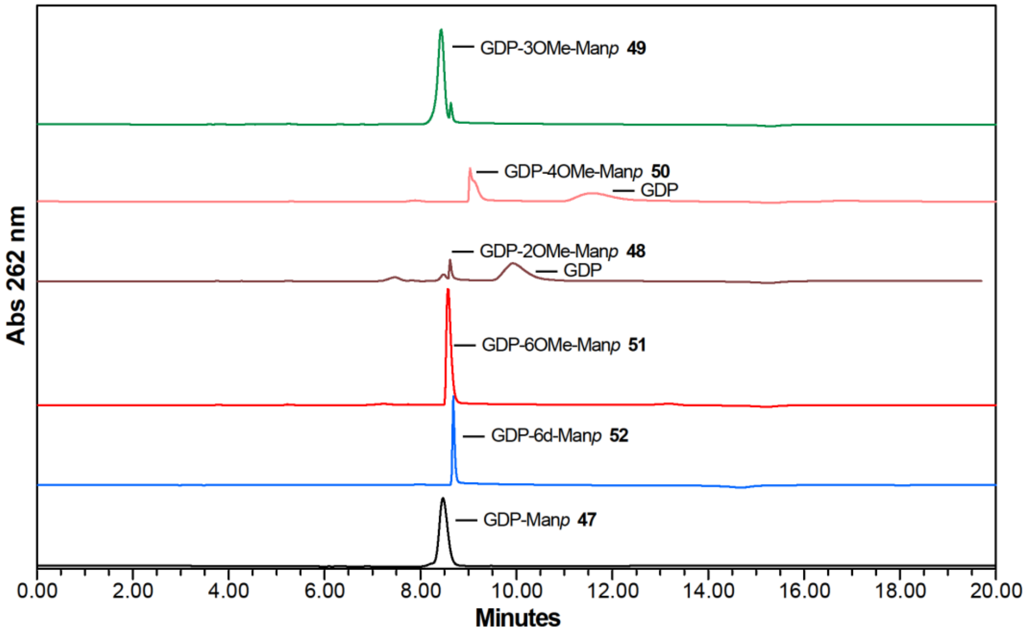


**Figure S2**. GDP-Man*p* analogue HPLC traces

## GDP-ManPP activity assay

The malachite green dye reagent was prepared as previously reported by MacMillan and coworkers [11]. Briefly, to a 100 mL volumetric flask were added 34.0 mg of malachite green oxalate salt, 1.236 g of ammonium molybdate, 3.4 mL of absolute ethanol, 80 mL of deionised water, 8.6 mL of concentrated HCl (37%), 1 mL of Tween 20, and deionised water up to 100 mL total volume. The resulting mixture was stirred for 1 h, then was filtered (0.2 μm) and stored at 4 °C for 7 days. The mixture was filtered again and calibrated by using a standard series of $KH_2PO_4$ solution at variable concentrations prior to use. In a 96-well plate, 25 μL of each standard was added to a well before the addition of 100 μL malachite reagent. The plate was incubated at 37 °C with mixing, and the absorbance was read at 650 nm over 10 min. The absorbance values at 10 mins were plotted against the phosphate concentration, and from linear fitting an equation was obtained, from which the slope gave the conversion factor.

Assays for GDP-ManPP activity were prepared containing Man*p*-1P or Man*p*-1P analogues (500 μM), GTP (2 mM), GDP-ManPP (50 ng), iPPase (0.5 U), $MgCl_2$ (8 mM), DTT (1 mM), in 25 μL of TRIS 50 mM pH 7.6. The reactions were incubated for 10 minutes at 37 ºC in a 96–well plate, after which time the reactions were quenched by the addition of 100 μL of malachite reagent. The colorimetric activity assay was carried out in a plate reader at 37 °C, with mixing, for 10 minutes and the absorbance at 650 nm was then read. Kinetic analyses were carried out under the same conditions as the activity assay, except variable concentrations of the Man*p*-1P or Man*p*-1P analogue substrates were used as listed in Table S1.

**Table S1:** Variable concentrations of six substrates used for kinetics analysis.

| **Man*p*-1P (46) (μM)** | **2-OMe (9) (μM)** | **3-OMe (10) (μM)** | **4-OMe (11) (μM)** | **6-OMe (12) (μM)** | **6-deoxy (13) (μM)** |
|---|---|---|---|---|---|
| 900.0 | 4000.0 | 4000.0 | 4000.0 | 1000.0 | 2000.0 |
| 450.0 | 2000.0 | 2000.0 | 2000.0 | 500.0 | 1000.0 |
| 225.0 | 1000.0 | 1000.0 | 1000.0 | 250.0 | 500.0 |
| 112.5 | 500.0 | 500.0 | 500.0 | 125.0 | 250.0 |
| 56.3 | 250.0 | 250.0 | 250.0 | 62.5 | 125.0 |
| 28.1 | 125.0 | 125.0 | 125.0 | 31.3 | 62.5 |
| 14.1 | 62.5 | 62.5 | 62.5 | 15.6 | 31.3 |
| 7.0 | 31.3 | 31.3 | 31.3 | 7.8 | 15.6 |
| 3.5 | 15.6 | 15.6 | 15.6 | | 7.8 |

# References


1. Bock, K.; Pedersen, C. *J. Chem. Soc., Perkin Trans. 2* **1974,** 293. doi:10.1039/p29740000293
2. Tam, P.-H.; Lowary, T. L. *Carbohydr. Res.* **2007,** *342*, 1741. doi:10.1016/j.carres.2007.05.001
3. Smith, F. *J. Chem. Soc.* **1951,** 2646. doi:10.1039/JR9510002646
4. Lemanski, G.; Ziegler, T. *Tetrahedron* **2000,** *56*, 563. doi:10.1016/S0040-4020(99)01053-4
5. Ottosson, H. *Carbohydr. Res.* **1990,** *197*, 101. doi:10.1016/0008-6215(90)84133-F
6. Esmurziev, A. M.; Simic, N.; Hoff, B. H.; Sundby, E. *J. Carbohydr. Chem.* **2010,** *29*, 348. doi:10.1080/07328303.2010.540055
7. Rozanas, C. R.; Gray, G. R. *Carbohydr. Res.* **1997,** *298*, 243. doi:10.1016/S0008-6215(96)00322-9
8. Haskins, W. T.; Hann, R. M.; Hudson, C. S. *J. Am. Chem. Soc.* **1946,** *68*, 628. doi:10.1021/ja01208a029
9. Sandström, C.; Hakkarainen, B.; Matei, E.; Glinchert, A.; Lahmann, M.; Oscarson, S.; Kenne, L.; Gronenborn, A. M. *Biochemistry* **2008,** *47*, 3625. doi:10.1021/bi702200m
10. Elling, L.; Ritter, J. E.; Verseck, S. *Glycobiology* **1996,** *6*, 591. doi:10.1093/glycob/6.6.591
11. Marchesan, S.; Macmillan, D. *Chem. Commun.* **2008,** 4321. doi:10.1039/b807016d
